# Synthesis, Characterization, and Cellular Uptake of Magnesium Maltol and Ethylmaltol Complexes

**DOI:** 10.1021/acsomega.1c04104

**Published:** 2021-10-28

**Authors:** Derek R. Case, Ren Gonzalez, Jon Zubieta, Robert P. Doyle

**Affiliations:** † Department of Chemistry, Syracuse University, 111 College Place, Syracuse, New York 13244, United States; ‡ Balchem Corporation, 52 Sunrise Park Road, New Hampton, New York 10958, United States

## Abstract

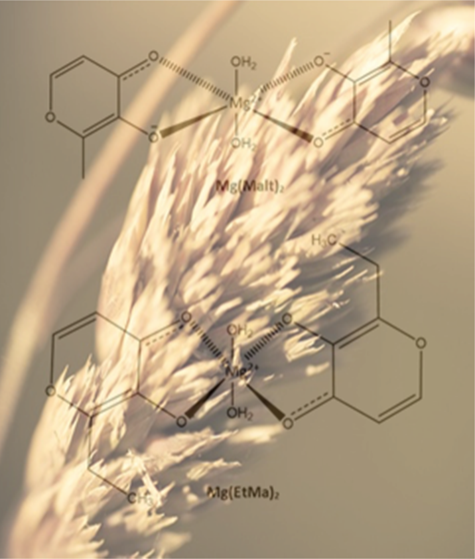

Magnesium deficiency and/or deficit (hypomagnesemia, <0.75 mmol/L in the blood) has become a recognized problem in healthcare and clinical settings. Concomitantly, supplementation has become recognized as the primary means of mitigating such deficiencies. Common magnesium supplements typically suffer from shortcomings: rapid dissociation and subsequent laxation (magnesium salts: e.g., magnesium chloride), poor water solubility (magnesium oxides and hydroxides), poor characterizability (magnesium chelates), and are/or use of non-natural ligands. To this end, there is a need for the development of fully characterized, water-soluble, all-natural magnesium compounds. Herein, we discuss the synthesis, solution and solid-state characterization, aqueous solubility, and cellular uptake of magnesium complexes of maltol and ethylmaltol, ligands whose magnesium complexes have yet to be fully explored.

## 1 Introduction

Latent magnesium deficiency (hypomagnesemia, defined as <0.75 mmol/L blood magnesium levels)^1^ is now considered a significant impactor of chronic disease.^2−5^ Tracking hypomagnesemia is complicated by the uneven distribution of magnesium in the human body and in particular by the low levels found in blood (<1% of total body magnesium).^2,6−16^ Correlations have, however, been developed between hypomagnesemia and a litany of chronic diseases affecting cardiovascular (arrhythmia, hypertension, etc.), bone (osteoporosis, etc.),^12−20^ neurological (migraines/headaches),^13^ and metabolic health (Type II Diabetes Mellitus (T2DM)).^14,21,22^

While there are multiple biological factors that impact total body magnesium levels, such as malabsorption in the lower gastrointestinal (GI) tract, and diseases associated with increased renal wasting (e.g., T2DM, alcoholism, etc.),^23^ it is believed that an inadequate intake of magnesium through diet is the predominant contributing factor.^23^ This inadequate intake is attributed primarily to diet^24−26^ but can be combated using oral magnesium supplementation.^19,20,22,27−30^

To date, the most ubiquitously used magnesium supplements have been salts (e.g., magnesium chloride, magnesium sulfate) and oxides/hydroxides of magnesium. While the oxides and hydroxides of magnesium retain the highest percent composition of magnesium, the effectiveness of these supplements is hindered by a lack of water solubility,^31^ which correlates with oral bioavailability.^1^ Interestingly, while the magnesium salts offer greater solubility, they are often subject to rapid dissociation and laxation and are excreted renally before most cellular uptake occurs.^10,32−34^ Additionally, many magnesium supplements are not fully characterized, which complicates dosing and the ability to translate such into pharmaceutical formulations.^35,36^ Poor flavor profile and non-natural ligands used also round out common issues with current magnesium supplements.

The naturally occurring compound maltol (IUPAC, 3-hydroxy-2-methyl-4H-pyran-4-one), found in malted grain, the Fraser Fir, or purple passionflower (*Passiflora incarnata*),^37−40^ among others, and the non-natural, but structurally related food additive ethylmaltol (IUPAC, 2-ethyl-3-hydroxy-4H-pyran-4-one) were selected as ideal magnesium chelate ligands for multiple reasons; both ligands have generally regarded as safe (GRAS) status,^38^ are water soluble (maltol, 1.2 g/100 mL;^39^ ethylmaltol, 5.84 g/100 mL),^40^ have the potential to serve as bidentate chelates to aid in complex stability, and exhibit a single monoanionic state and a weakly alkaline character resulting in a pH-buffering capacity that is ideal for the upregulation of claudins (the primary magnesium transporter) within the passive paracellular uptake pathway.^33^ In addition, both compounds possess a caramel-like smell and taste that is attractive when considering supplements for oral ingestion.

Syntheses of magnesium maltol and magnesium ethylmaltol were conducted in water, and the compounds isolated were analyzed in the solution and solid state. Complex water solubilities were also investigated. Cellular uptake was evaluated utilizing a human colorectal carcinoma (CaCo-2) cell line, a common *in vitro* model for the lower intestine, whereupon the majority of magnesium uptake occurs. Analyses indicate the successful synthesis of 6-coordinate, octahedral magnesium complexes in a 1:2 Mg/maltol (1) and a 1:2 Mg/ethylmaltol (2)–*bis*-bidentate chelate arrangement, with open coordination sites occupied by water.

## 2 Results and Discussion

### 2.1 Synthesis of **1** and **2**

Both **1** and **2** were synthesized from a magnesium oxide starting material in the presence of citric acid to aid in the solubility of the relatively water-insoluble metal oxide; the citric acid provides a proton source. Addition of citric acid at 0.25 equiv was the lowest concentration found that could drive the reaction while also minimizing the formation of magnesium citrate, with ^1^H NMR of both **1** and **2** indicating 7.2 and 6.1% magnesium citrate in the final products, respectively (Figures S2–S4). Increasing the equivalents of magnesium oxide to 1.2 equiv and 1.1 equiv for the synthesis of **1** and **2**, respectively, was required to push the stoichiometric yield of the product and negate the return of unreacted starting materials (data not shown). Specifically, at 1:2 equiv of magnesium oxide/maltol, upon cooling the solution from reaction temperature, a white precipitate was observed. The analysis of the dried precipitate via EA (Figure S1) confirmed it to be unreacted maltol. Given the requirement to have citric acid present to drive the reaction, minimized as it is to 0.25 equiv, it is clear that the citrate is outcompeting maltol for magnesium binding. Thus, an additional stoichiometric amount of MgO is necessary to drive complete chelation of all maltol starting materials.

### 2.2 Structural Characterization of **1** and **2** via Infrared Spectroscopy

**Figure 1:**
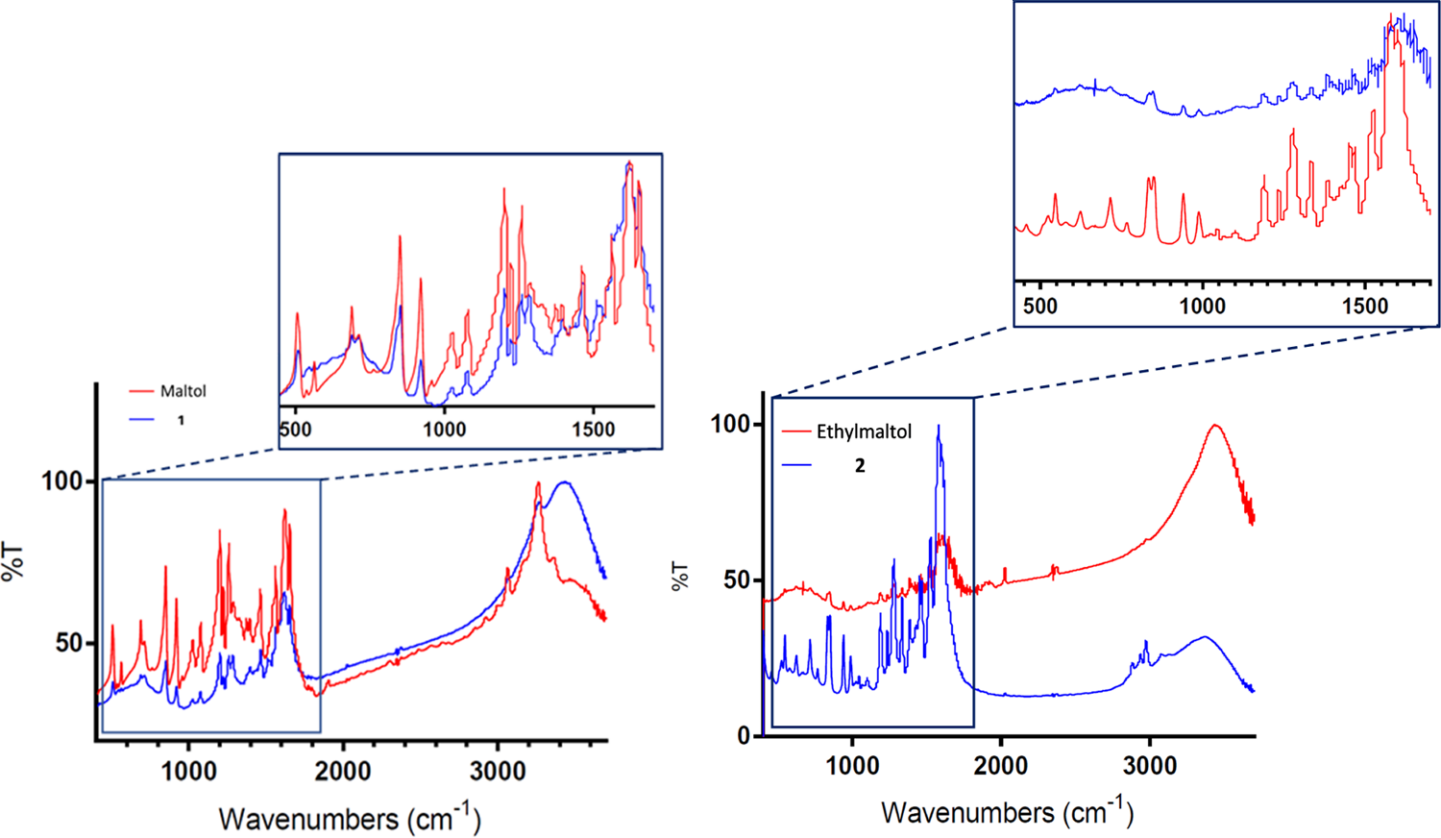
FT-IR spectra of maltol and 1 (left) and ethylmaltol and 2 (right); insets at the right are a zoomed display of the region between 500 and 1700 cm^–1^ to emphasize changes observed in the region attributed to the ketone fingerprint (1500–1700 cm^–1^).

The infrared spectra of **1** and **2** were compared to the infrared spectra of both maltol and ethylmaltol, respectively (Figure 1). Fourier transform infrared radiation (FT-IR) of both ligands showed changes to the frequency regions that corresponded specifically to the −OH stretching mode of both ligands associated with the coordination of this moiety. There is a slight change observed in the frequency of the signals attributed to the ketone moiety of **1** to higher energy relative to maltol.^41^

This suggests magnesium coordination about the ketone, and a shift to slightly higher energy is consistent with magnesium coordination as reported by Nara et al.^42^ However, this is different than the observed signal shifts observed for other divalent metal–maltol complexes such as *bis*maltolato zinc (II).^43^ Upon coordination to zinc, the infrared maltol signals attributed to the ketone moiety are shifted to lower energy. This may be the result of zinc being less electropositive in character than magnesium, thus resulting in less ionic character upon coordination, but may also be attributed to differences in ionic radii of the two metals. No significant change to the region associated with the ketone is observed for **2**. However, coordination about this site is again supported by ^13^C NMR. Additionally, the spectra of both **1** and **2** indicate the presence of coordinated water signified by broad signals between 3200 and 3500 cm^–1^, as were observed for the previously described zinc maltol complexes.^43^ Further insight into the conclusions drawn from the FT-IR spectra is provided in Table 1 and Supporting Information Figures S5 and S6.

**Table 1:** Assignment of the Infrared Spectra Values of Maltol, **1**, Ethylmaltol, and **2**. Additional Shifting upon Ligand Coordination to Magnesium is Also Provided

complex	IR frequency (cm^–1^)	assignment	change (cm^–1^)
maltol	3260	ν(OH), C–OH	
	1655	ν(C=O)	
	1621	ν(C=O)	
**1**	3435	v(OH), H_2_O	
	3264	ν(OH), C–OH	4
	1655	ν(C=O)	
	1617	ν(C=O)	4
ethylmaltol	3369	ν(OH), C–OH	
	1617	ν(C=O)	
	1525	ν(C=O)	
**2**	3447	ν(OH), H_2_O	

### 2.3 Determining Degree of Hydration of **1** and **2** via Thermal Analysis

**Figure 2:**
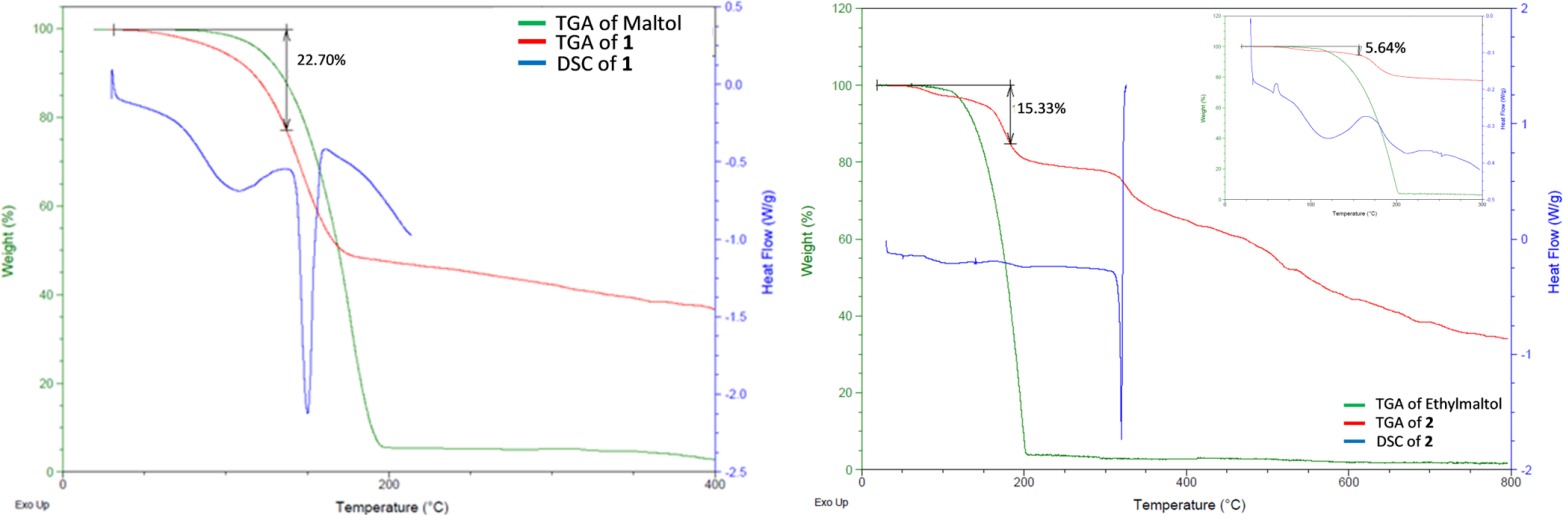
TGA/DSC of both **1** (top) and **2** (bottom) indicating the observed percent weight changes of each complex and the thermodynamic events that correspond to the observed weight changes. The inset highlights the first endotherm attributed to the loss of water.

The thermal analysis of **1** was conducted relative to maltol. Maltol exhibited a continuous percent weight loss onset at ∼70 to 200 °C and stopped decreasing in percent weight at approximately 5%, thus suggesting decomposition of maltol between 160 and 200 °C, which is consistent with the known melting point of maltol at 160 °C.^44^ Thermogravimetric analysis (TGA) analysis of **1** exhibited a similar decomposition trend differing only with the percent weight loss exhibited by **1** reaching a minimum at approximately 40%. The differential scanning calorimetry (DSC) spectrum of **1** exhibited two endotherms: a broad endotherm with an apex at approximately 120 °C attributed to the loss of coordinated water from complex **1** and a secondary more intense, sharper endotherm attaining apogee at approximately 160 °C. This endotherm is attributed to the thermal decomposition of the maltol ligand (Figure 2), which is consistent with the TGA of maltol. The endotherm at 120 °C corresponds to a percent weight decrease of 22.70% observed on the TGA of **1**, which is attributed to the loss of four water molecules given a predicted percent weight change of 20.80%. While the EA of **1** suggests only three waters, this difference is attributed to different hydrated states given the propensity of magnesium to take on water.^45,46^

As observed with maltol, ethylmaltol exhibited only one continuous percent weight decrease from approximately 70 to 200 °C and stops decreasing in weight at approximately 5% weight (Figure 2). This profile is attributed to the thermal decomposition of the ethylmaltol ligand, which is predicted to be roughly the same as maltol at ∼160 °C. The TGA of **2** differed from that of ethylmaltol in that it exhibited two distinguishable percent weight decreases and stopped decreasing in percent weight at approximately 35%. Both percent weight changes correspond to two separate endotherms observed on the DSC of **2**, one broad endotherm apexed at approximately 110 °C and a secondary sharp, and substantially more intense, endotherm with an apex at approximately 320 °C. The first broad endotherm observed on the DSC of complex **2** shows a corresponding percent weight change of 15.33%, which corresponds to the loss of three waters from the overall [Mg(EtMa)_2_(H_2_O)_2_]·H_2_O] complex supported by the EA with a predicted weight percent change of 15.15%. The secondary, more intense, endotherm at approximately 320 °C is attributed to the decomposition of the ethylmaltol ligand. The number of waters observed for **1** via thermal analyses predicts two waters directly coordinated to the magnesium core and two additional waters of crystallization. The presence of three waters is consistent with EA. However, magnesium readily absorbs water and differing drying conditions and/or sample preparations likely have contributed to the different hydration states noted.^30,46^ The three waters observed for **2** support two coordinated waters and one water of crystallization.

### 2.4 Structural Characterization of **1** and **2** via One-dimensional (1D) and Two-dimensional (2D) ^1^H/^13^C NMR

Mg^2+^ readily coordinates with hard Lewis bases as exemplified by the monodentate magnesium chelates of formic acid,^47^ orotic acid,^48^ maleic acid,^49^ the bidentate magnesium chelates of mandelic acid and malic acid,^50^ and the tridentate magnesium chelate of citric acid.^51−53^ Ligand chelation to the divalent magnesium cation is often characterized by an observable shift in the NMR, or a change in signal resolution, of the proton signals adjacent to the Lewis bases of the ligand, due to the electropositive character of the metal.^54^^–60^ Given the impact that concentration may have on the shifting of proton and carbon signals, each sample of **1** and **2** was analyzed at equimolar concentrations to maltol and ethylmaltol, respectively, with the instrument internally calibrated to TMS and each spectrum calibrated to the residual HOD peaks present in the D_2_O solvent.

**Figure 3:**
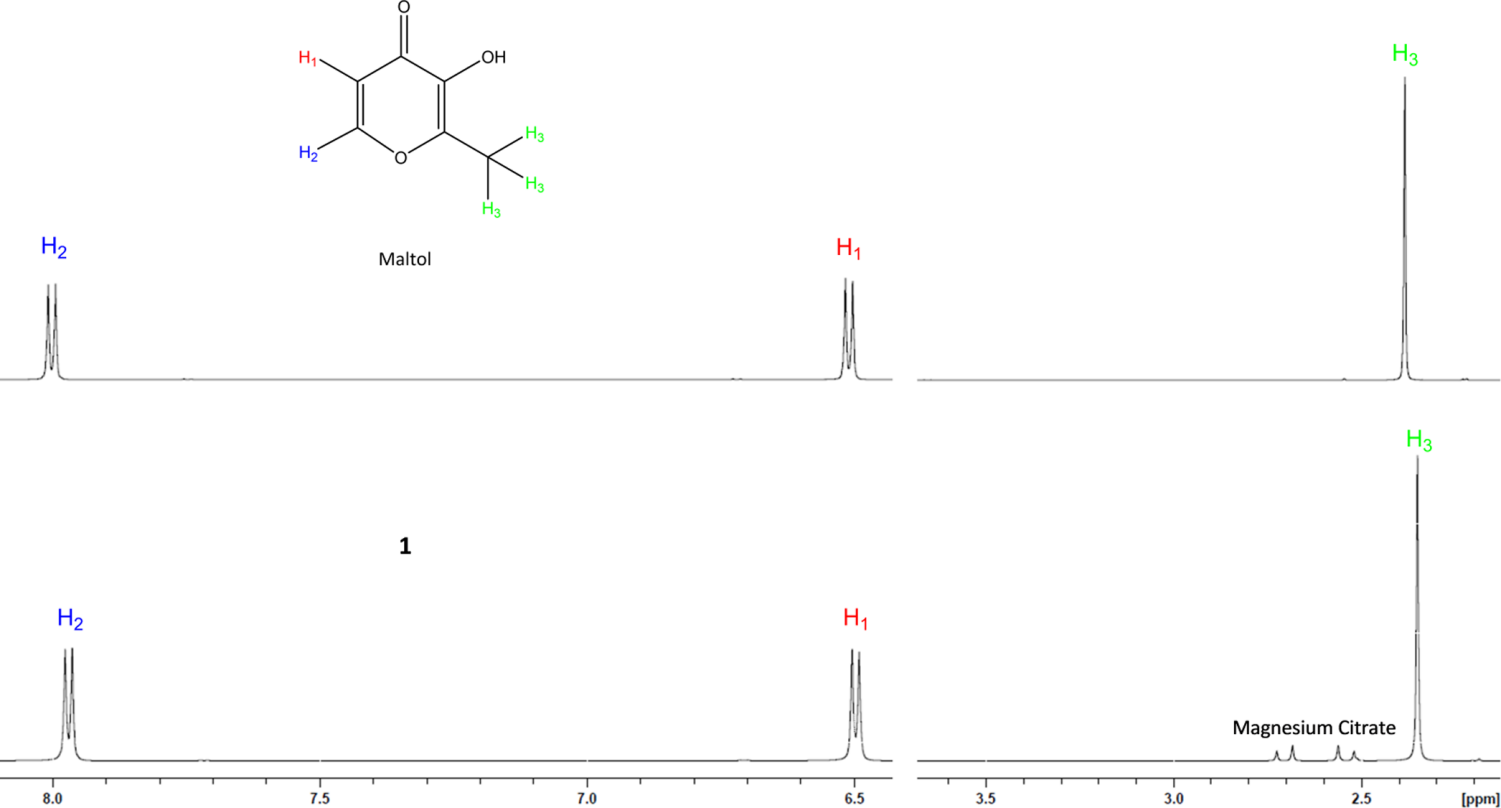
Proton NMR overlay of maltol (t**op**) and **1** (b**ottom**) conducted in D_2_O showing the observable upfield shifts of all protons and the subsequent change in the proton splitting pattern. Solutions were analyzed at an equimolar concentration. Full spectra are available in Supporting Information Figures S7–S10.

^1^H NMR was conducted on both maltol and **1** in 700 μL of D_2_O (Figure 3). At equimolar concentrations, the integration of maltol and **1** is conserved (Figures S9 and S10). Additionally, **1** showed a small but observable upfield shift for all three protons of 0.03 ppm for H_2_, 0.01–0.02 ppm for H_1_, and 0.03 ppm for H_3_.

**Figure 4:**
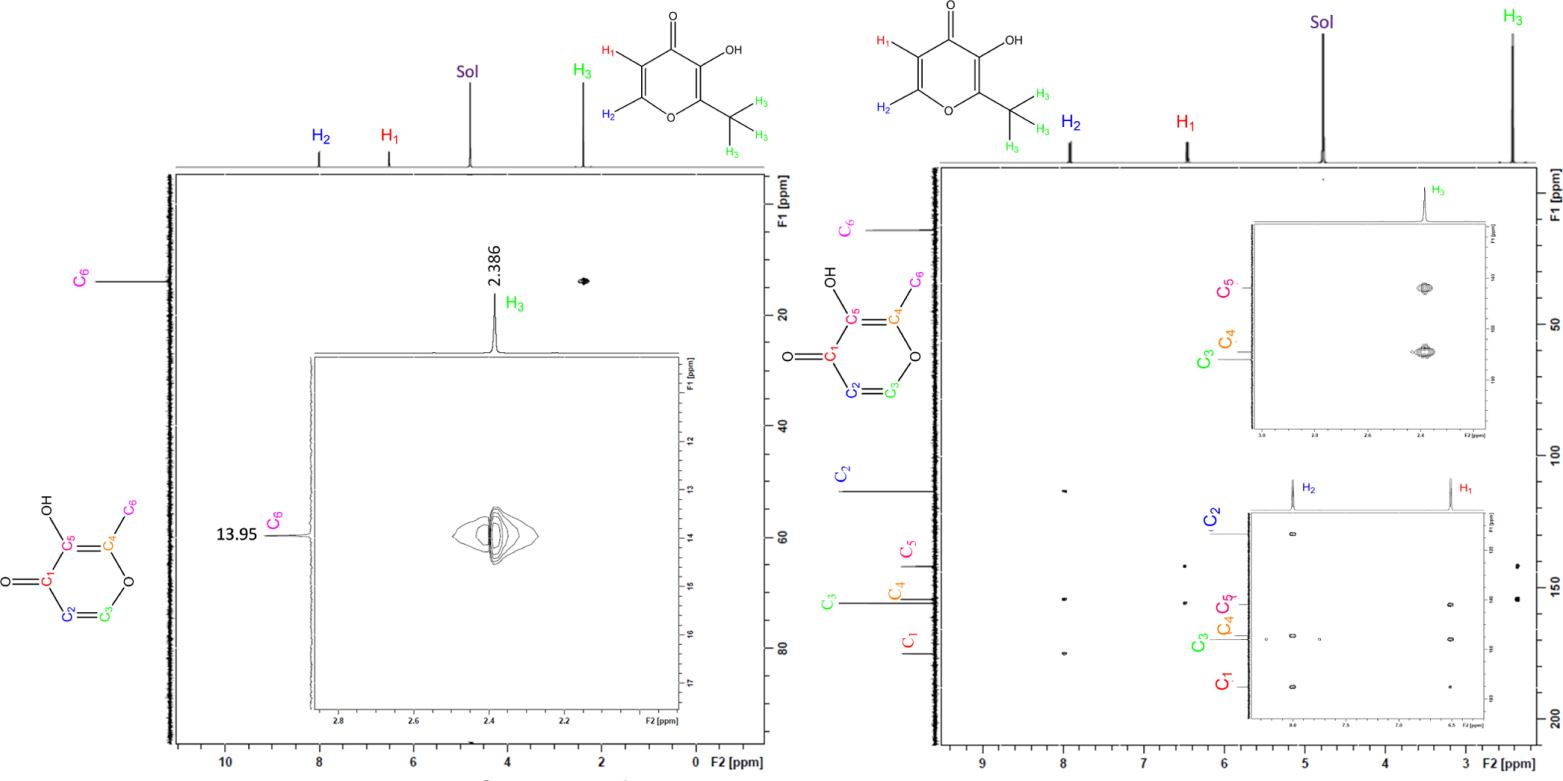
2D HSQC of maltol (l**eft**) showing a single coherence point between each proton and its corresponding carbon (1:1) and the 2D HMBC of maltol (r**ight**) showing three correlation points between H_2_ and three carbons (C_1_, 175.1 ppm; C_4_, 154.5 ppm; and C_2_, 113.40 ppm), H_1_ and two carbons (C_5_, 142.0 ppm and C_3_, 156.0 ppm), and two correlation points between H_3_ and two carbons (C_5_, 142.0 ppm and C_4_,154.5 ppm) (3:2:2). NMR was conducted in D_2_O. Full spectra are available as Supporting Information Figures S12–S15. Sol = solvent HOD. Solutions were analyzed at an equimolar concentration.

**Figure 5:**
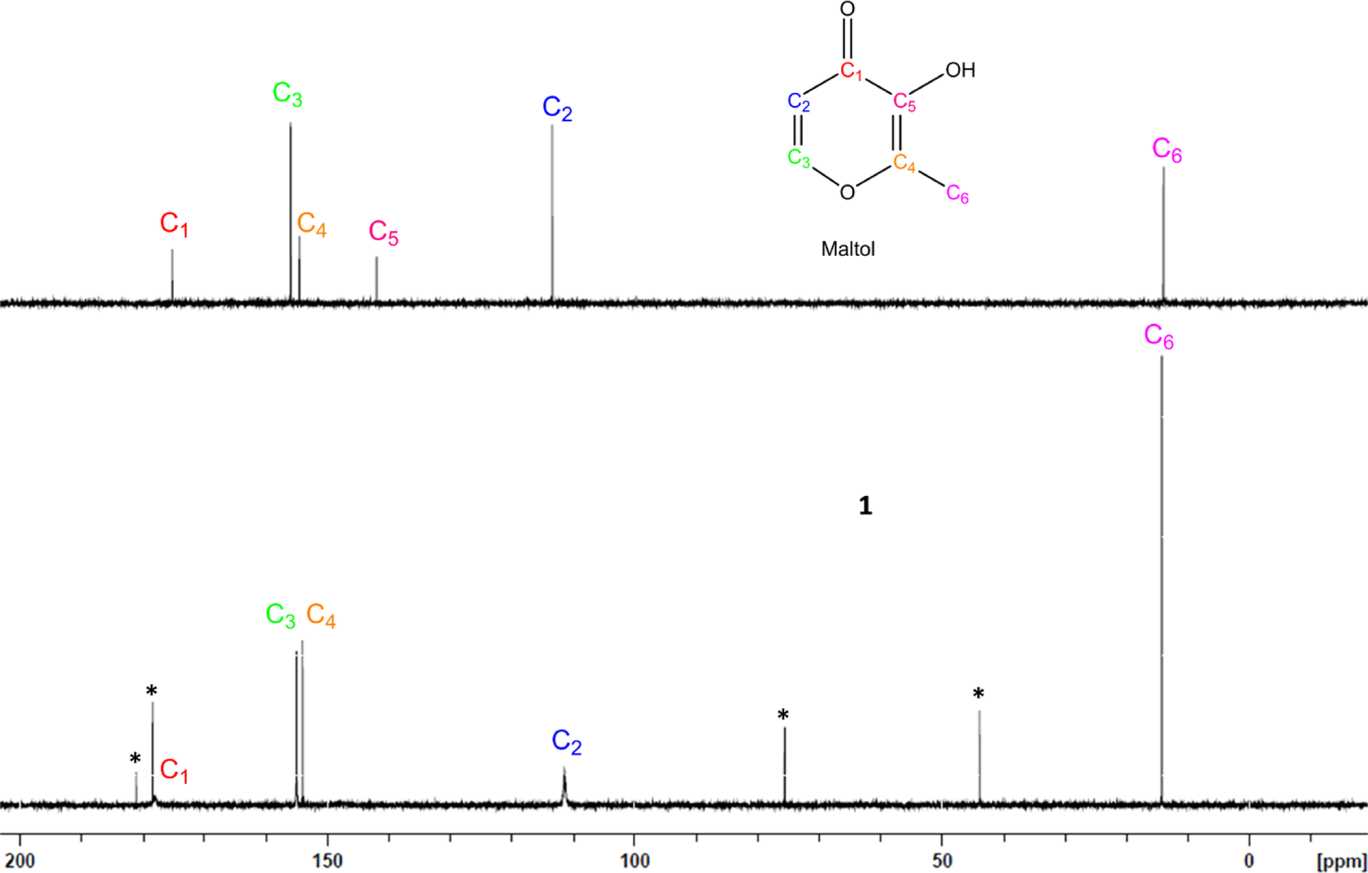
^13^C NMR overlay of maltol (**top**) and magnesium maltol (**1**, **bottom**) conducted in D_2_O. ^13^C NMR shows a significant reduction in the intensity of the C_1_ and C_2_ carbon signals for complex **1**, as well the disappearance of the C_5_ signal. Solutions were analyzed at an equimolar concentration. * Indicates peaks attributed to magnesium citrate.

**Figure 6:**
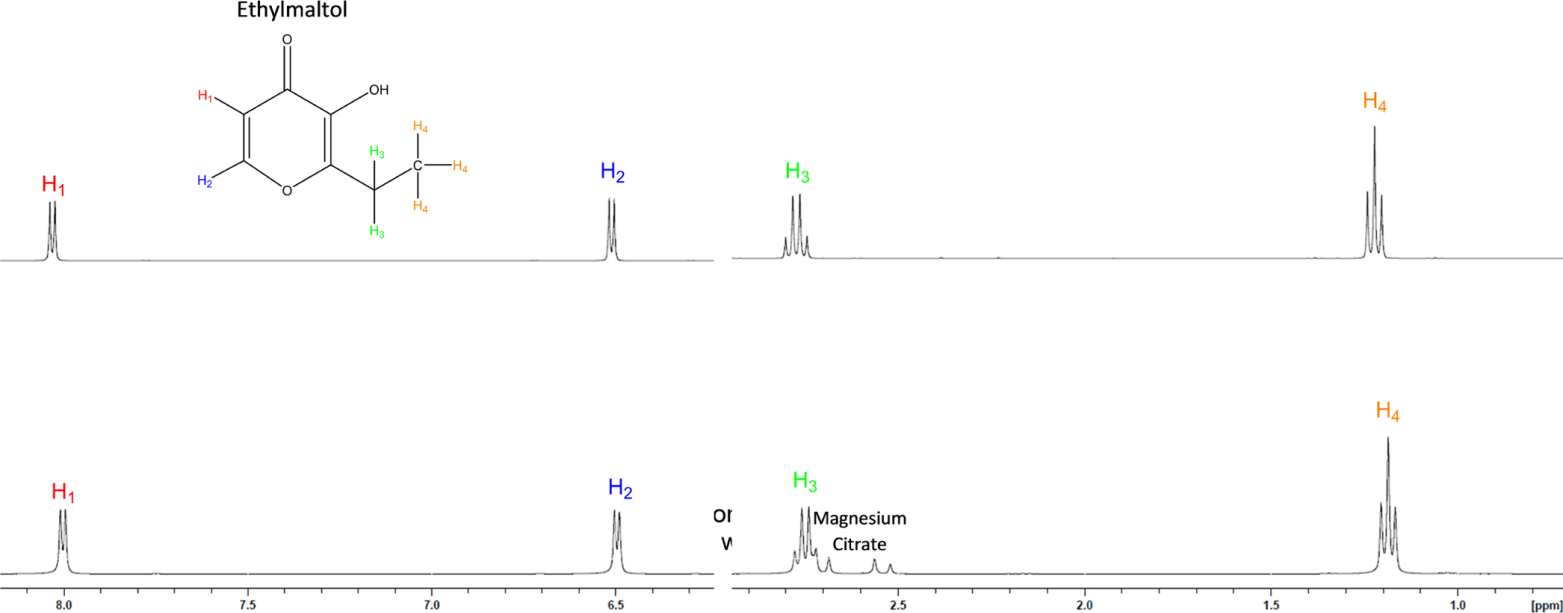
Proton NMR overlay of ethylmaltol (top) and **2** (bottom) conducted in D_2_O showing the observable upfield shifts of all protons and the subsequent change in the proton splitting pattern. Solutions were analyzed at an equimolar concentration.

**Figure 7:**
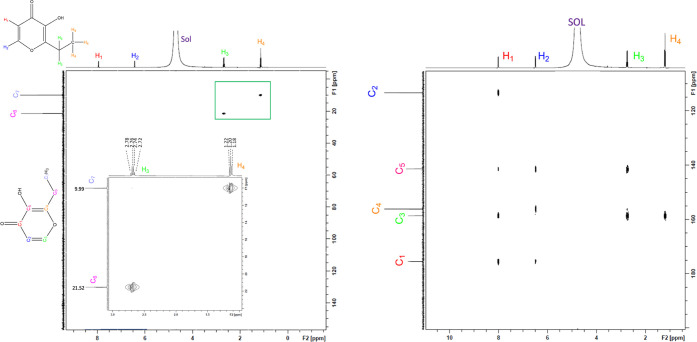
2D HSQC of ethylmaltol (l**eft**) showing a single coherence point between each proton and its corresponding carbon (1:1) and the 2D HMBC of ethylmaltol (r**ight**) showing three correlation points between H_2_ and three carbons (C_1_, 175.6 ppm; C_3_, 158.6 ppm; and C_5_, 141.4 ppm), H_1_ and four carbons (C_2_, 113.1 ppm; C_5_, 141.4 ppm; C_3;_ 158.5 ppm; and C_1_, 175.6 ppm), two correlation points between H_3_ and two carbons (C_5_, 141.4 ppm and C_4_, 156.4 ppm), and one correlation point between H_4_ and one carbon (C_3_, 158.5 ppm) (3:2:2:1). NMR was conducted in D_2_O. SOL = solvent HOD. Solutions were analyzed at an equimolar concentration.

**Figure 8:**
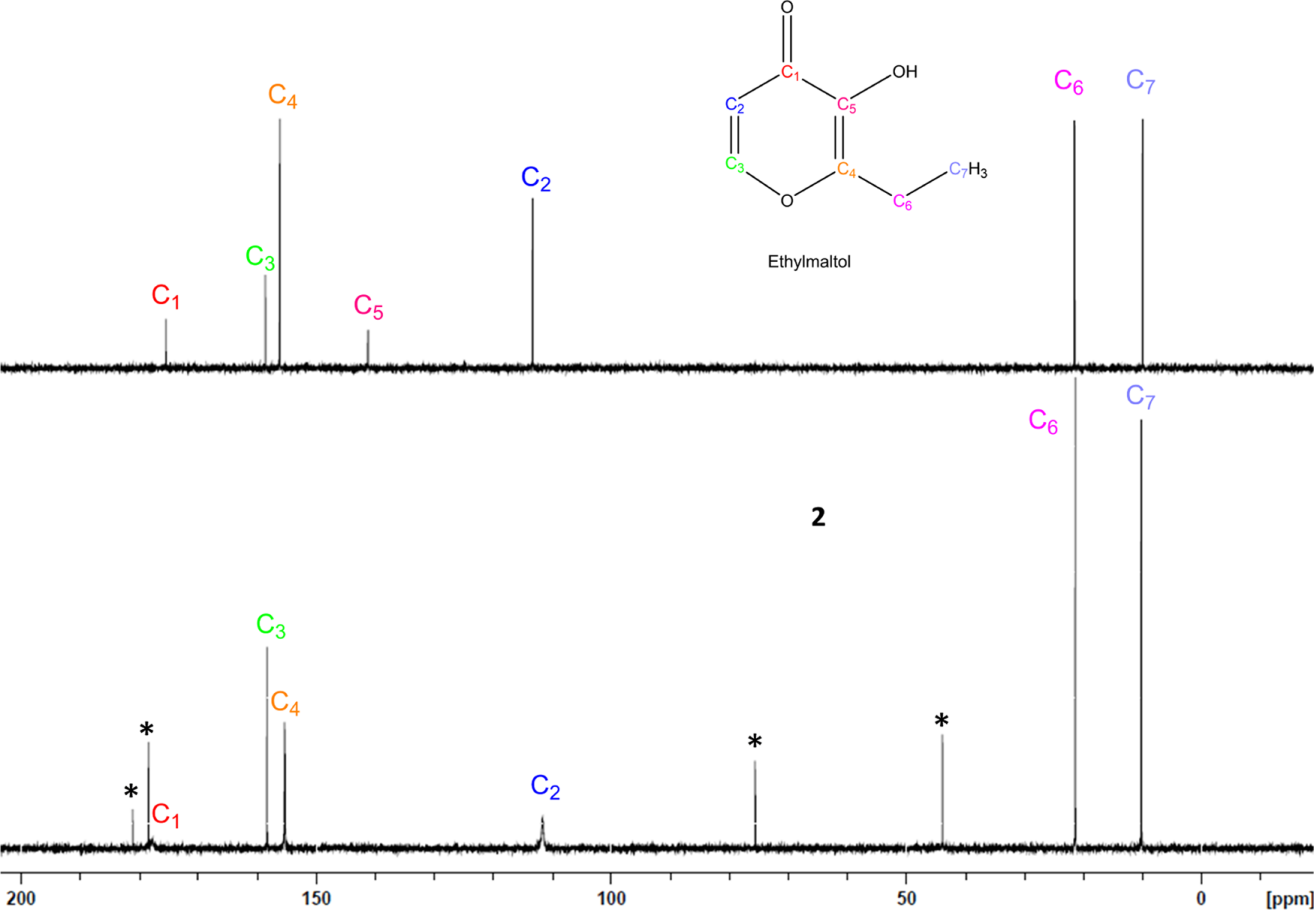
^13^C NMR overlay of ethylmaltol and magnesium ethylmaltol conducted in D_2_O. ^13^C NMR shows a significant reduction in the intensity of the C_1_ and C_2_ carbon signals for complex **2**, as well the disappearance of the C_5_ signal. Solutions were analyzed at an equimolar concentration. The full spectra ^13^C NMR with ppm shift are provided in Supporting Information Figure S25. * Indicates peaks attributed to magnesium citrate.

Both heteronuclear single quantum coherence (HSQC) and heteronuclear multiple bond correlation (HMBC) confirmed the proton and carbon signal assignments of maltol, showing that C_1_ (Figure 4) was the most downfield carbon signal at 175.20 ppm, while C_5_ was assigned at 154.50 ppm and C_2_ was assigned at 113.40 ppm. Evaluation of maltol ^13^C NMR (see Supporting Information Figure S11) comparatively to **1** showed a significant reduction in intensity, as well as broadening of the C_1_ and C_2_ carbon signals. The analysis also showed a complete disappearance of the signal attributed to C_5_ (Figures 5 and 6); a similar trend was observed for the ^13^C NMR of **2**, except for C_5_ peak intensity (Figures 7 and 8). Additionally, there was an observable downfield shift of the C_1_ carbon signal (178.20ppm) and an upfield shift of the C_2_ (112.40 ppm) carbon signal (full HSQC/HMBC spectra of maltol are available as Supporting Information Figures S12 and S13 and full HSQC/HMBC spectra of **1** are available as Supporting Information Figures S14 and S15).

^1^H NMR was conducted on ethylmaltol and the pure and dried **2** in D_2_O. At equimolar concentrations, the integration of ethylmaltol and **2** is conserved (Supporting Information Figures S16 and S17). Additionally, **2** showed a small observable upfield shift for each of the proton peaks of 0.01 ppm, 0.01, 0.01, and 0.01 ppm for H_1_–H_4_, respectively (Figure 6), a trend similar to that noted for **1**. The full spectra are available as Supporting Information Figures S16–S19. Assignments of all proton and carbon signals were confirmed via 2D ^1^H–^13^C NMR (full spectra are available as Supporting Information Figures S20–S25).

### 2.5 Evaluating the Solubility of **1** and **2**

The solubility of 1 and 2 was determined at room temperature. Over triplicate independent runs, the solubility of 1 was found to be 15.6 ± 1.17 g per 100 mL of H_2_O, and the solubility of 2 was found to be 16.2 ± 0.75 g per 100 mL of H_2_O. The solubility of 1 is approximately 13X greater than that of maltol (1.2 g/100 mL) and the solubility of 2 is approximately 2.8X greater than that of the ethylmaltol ligand (5.84 g/100 mL) (Table 2). These solubilities are consistent with the reported solubilities of maltol and ethylmaltol, as described by Liu et al.^39^ and Li et al.^55^ The solubilities of 1 and 2 are greater than their organic counterparts, and this is attributed to inherent magnesium aquation. It may also be attributed to the more ionic nature of the overall compound relative to the standalone ligands.

**Table 2:** Solubility of **1** and **2** Compared to the Commonly Used Magnesium Supplements MgO and MgCl_2_

complex name	molecular weight (g/mol)	core formula	%Mg in compound	solubility (g/100 mL)	refs
magnesium chloride Hexahydrate	95.2	MgCl_2_·6H_2_O	11.9	54	(48)
magnesium oxide	40.3	MgO	60.3	0.010	(31)
**1**	310.5	Mg(C_6_H_5_O_2_)_2_(H_2_O)_2_	7.8	15.6 ± 1.17	*
**2**	338.6	Mg(C_7_H_7_O_3_)_2_(H_2_O)_2_	7.2	16.2 ± 0.75	*

### 2.6 Cellular Uptake of **1** and **2**

**Figure 9:**
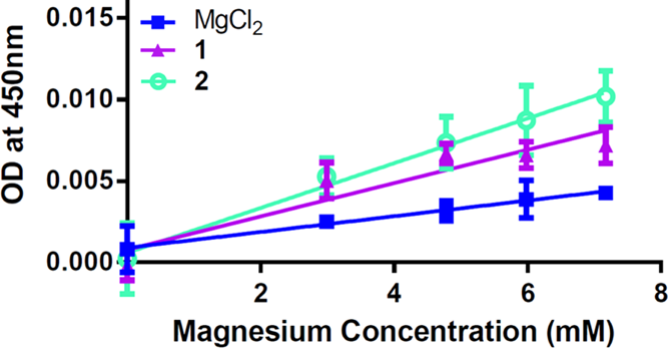
Cellular uptake of MgCl2, **1**, and **2** in CaCo-2 cells. Slope/*R*^2^ MgCl2: 5 × 10^–5^/0.7373. Slope/*R*^2^**1**, 0.0001/0.8198. Slope/*R*^2^**2**, 0.0001/0.8433. SEM, MgCl2 = ±0.0006, **1** = ±0.001, **2** = ±0.001; Upper 95% C.I., MgCl_2_ = 0.004, **1** = 0.008, **2** = 0.011; and Lower 95% C.I. = MgCl_2_ = 0.001, **1** = 0.001, **2** = 0.001.

Cellular uptake of **1** and **2** was conducted in CaCo-2 cells at an incubation time of 40 min (Figure 9). Uptake was evaluated with the understanding that both **1** and **2** contained ∼6–7% magnesium citrate, and the concentrations are based upon a total concentration of magnesium contributed from both species as based upon molecular weight. Both compounds provided substantial uptake of magnesium, with **2** showing slightly greater uptake than **1**, at a lower percent magnesium composition (7.2 versus 7.8%, respectively).

The uptake of **1** and **2** is similar, consistent with given similarities in solubility and the percent composition of magnesium of **1** and **2**. Future uptake studies will be conducted at solution saturation in vitro and in vivo.

### 2.7 Conclusions

Hypomagnesemia is a greatly underappreciated clinical issue and is common in critically ill patients, where it may lead to complications, from severe to fatal. Development of magnesium compounds that are fully characterized and that have the properties and benefits of being readily water soluble, all-natural/GRAS and readily absorbed is a current unmet need. Such compounds not only offer ready incorporation into supplements but also have scope to become magnesium pharmaceuticals, which can be used in a clinical setting to offset side effects of magnesium deficiency such as cardiovascular and neuromuscular manifestations. Herein, we describe the syntheses of magnesium maltol (**1**) and magnesium ethylmaltol (**2**). Solution-state and solid-state characterization enabled full characterization of both complexes, and analysis of cellular uptake data in the human CaCo-2 cell line confirmed cellular entry. Given the characterization, water solubility, cellular uptake, and all-natural/GRAS status of the ligands (magnesium oxide and citric acid starting materials), these compounds offer great scope for future development as food/supplement ingredients and/or for pharmaceutical purposes.

## 3 Experimental Section

### 3.1 Materials

Magnesium oxide (99.99% metals basis) was purchased from Fisher Scientific. Maltol (≥99.0%, food chemicals codex (FCC), food grade (FG)), ethylmaltol (≥99.0%, FCC, FG), citric acid (ACS Reagent, ≥99.5%), D_2_O and dimethyl sulfoxide (DMSO)-*d*6 NMR solvents, potassium bromide (KBr), and magnesium chloride hexahydrate (BioReagent, ≥97.0%) were purchased from Sigma-Aldrich (St. Louis, MO). Deionized water was obtained in-house. Colorimetric assay kits for magnesium uptake quantification were purchased from BioVision (Catalog #385-100; Milpitas, CA). Stock solutions for magnesium uptake assays were made in-house. CaCo-2 (HTB-37TM) cells, Dulbecco’s Modified Eagle Medium (DMEM) (30-2002), and magnesium/calcium-free Hank’s Balanced Salt Solution were purchased from ATCC (Manassas, VA). Hank’s Balanced Salt Solution (HBSS) and fetal bovine serum (FBS) were purchased from Gibco (Waltham, MA). T-25cm^2^ culturing flasks were purchased from Avantor (Radnor, PA). Clear 96-well assay plates were purchased from ThermoFisher (Waltham, MA).

### 3.2 Experimental Method

Electrospray ionization mass spectrometry (ESI-MS) was carried out on a Shimadzu 8040 liquid chromatography tandem-mass spectrometry (LC-MS/MS); samples were analyzed utilizing a solvent system of H_2_O/MeOH/0.1% trifluoroacetyl (TFA) at a flow rate of 0.2 mL/min over a 1.5 min time frame and evaluated from 0 to 600 m/z. 1D- and 2D-NMR were conducted on a Bruker Avance III HD 400 MHz instrument; each analyzed sample of **1** and **2** relative to maltol and ethylmaltol, respectively, was conducted at an equimolar concentration. The NMR instrument is internally calibrated to TMS (ppm = 0) and each reported spectrum is further calibrated to the residual HOD signal present in the D_2_O analytical solvent system. Each ^13^C NMR was obtained utilizing 1024 scans. FT-IR was carried out on a Nicolet Infrared Spectrophotometer. TGA was carried out on a TA Instrument Q500 from 20 to 800 °C. DSC was carried out on a TA Instrument Q2000 from 30 to 400 °C. Elemental analysis (EA) was conducted by Intertek Pharmaceutical Services (Whitehouse, NJ). Solubility of **1** and **2** was conducted at room temperature and evaluated by adding small amounts of material to 1 mL of volume until observed saturation; the sample of known mass was then massed again and the difference was calculated as the soluble fraction. Uptake of **1** and **2** in CaCo-2 cells was determined on a FlexStation 3 (Molecular Devices). Cellular uptake data was plotted using Graphpad Prism 8 software.

### 3.3 Culturing of CaCo-2 Cells

CaCo-2 cells were taken from liquid N_2_ stocks and rapidly thawed using a water bath at 37 °C. Cryopreservation media was removed with a micropipette after cells were pelleted via centrifugation for 2 min at 125 g. Cells were resuspended in 1 mL of Dulbecco’s Modified Eagle Medium (DMEM) that had been incubated at 37 °C and cultured in DMEM (total volume of 5 mL) with a seeding density of 3.6 × 10^4^ cells/cm^2^ in a T-25 cm^2^ culture flask and left to grow in an incubator at 37 °C and 5% CO_2_. When cultures reached 90%+ confluency, cells were detached with manual scraping and gentle agitation and pipetted. Two T-25 cm^2^ culturing flasks were combined and centrifuged into a pellet for 2 min at 125 g; the old media was pipetted off and cells were resuspended in 11 mL of fresh DMEM. Cells were plated in a 96-well plated at 100 μL/well and left to grow to 90%+ confluency to form a monolayer. Plated cells were used to determine magnesium uptake.

### 3.4 Synthesis of Magnesium Maltol (1), Scheme 1

**Scheme 1:**
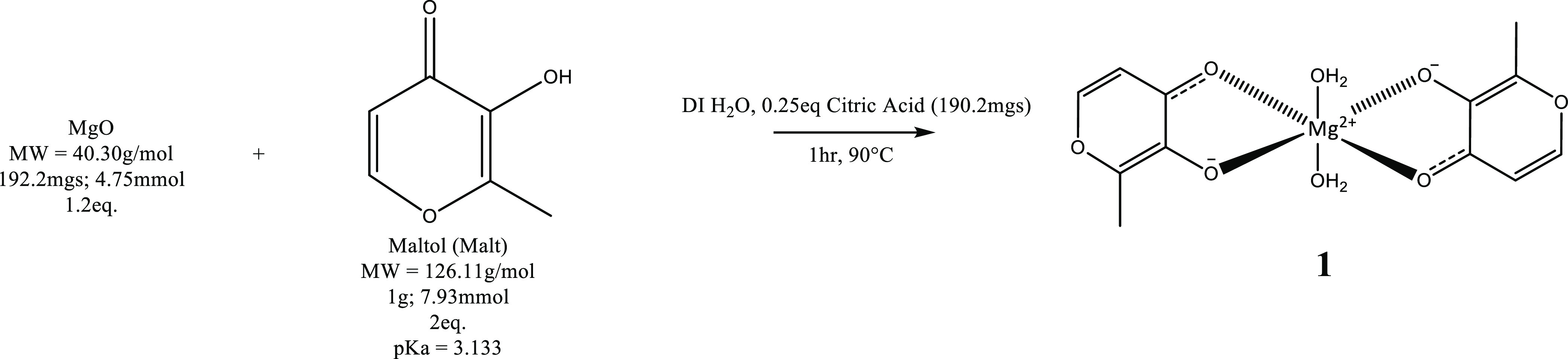
Synthesis of **1** Synthesis was conducted at a 1.2:2 molar equivalent of MgO and maltol. Citric acid (CA, 0.25 equiv) was added to provide a proton source for magnesium oxide

A 1.00 g sample of maltol (7.93 mmol; 2 equiv) was dissolved in 10 mL of DI H_2_O in a 50 mL round-bottom flask, with constant stirring at 90 °C (Scheme 1). A separate solution of 192.2 mg of magnesium oxide (MgO, 4.75 mmol; 1.2 equiv) was taken up in 10 mL of H_2_O, with the addition of 190.2 mg of citric acid (CA, 0.25 equiv), constantly stirred and heated to 90 °C. The MgO/CA solution was subsequently added to the maltol solution in small increments over ∼5 min. Upon addition, the mixture was a translucent white color that solubilized in about 30 s; each subsequent addition was administered when the previous addition had become wholly soluble. After all additions, the reaction was noted as colorless and clear. The reaction was conducted for 1 h, whereupon the solution was noted as yellow and clear. The reaction was allowed to cool to room temperature and the pH was noted as 7.80. The solution was dried *in vacuo*, producing a tan solid, which was used for subsequent analyses. The yield of **1** was stoichiometric relative to maltol with a purity of 92.8% based on ^1^H NMR. The solubility of **1** was determined to be 15.6 g/100 mL H_2_O. 1H NMR (D_2_O, 400 MHz): δ 4.79 (s, 1H), 7.95–7.94 (d, 1H, H2, *J* = 5.26 Hz), 6.48–6.46 (d, 1H, H1, *J* = 5.38 Hz), 2.33 (s, 1H, H3). EA: Theo for {[Mg(C_6_H_5_O_3_)_2_(H_2_O)_2_].H_2_O}: C = 43.17%, H = 4.75%; Exp: C = 43.33%, H = 4.49% (Figure S1)

### 3.5 Synthesis of Magnesium Ethylmaltol (2), Scheme 2

**Scheme 2:**
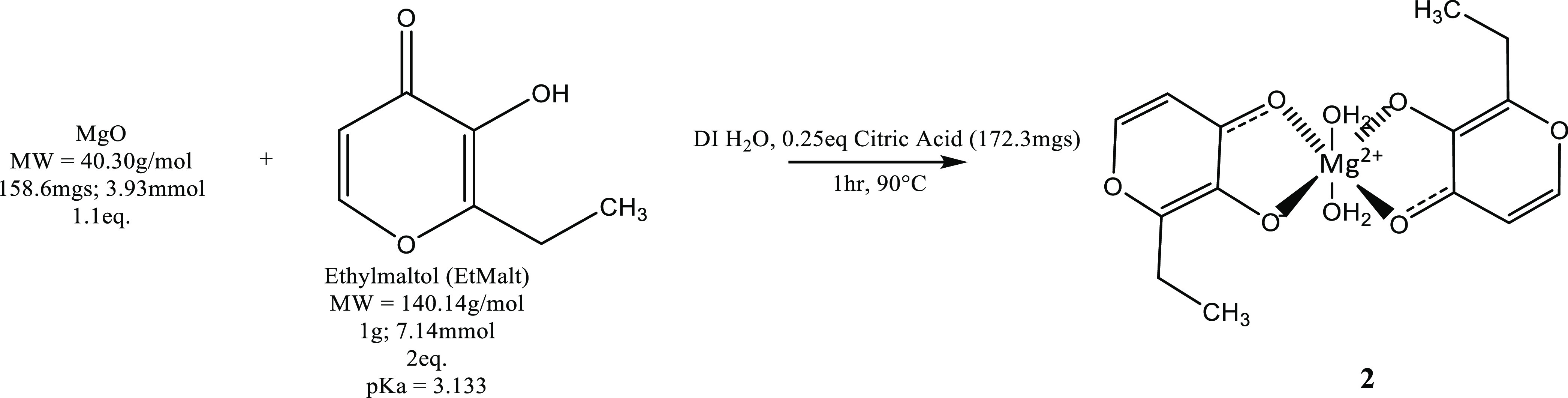
Synthesis of **2** Synthesis was conducted at 1.1:2 molar equivalents of MgO:ethylmaltol. Citric acid (CA, 0.25 equiv) was added to provide a proton source for magnesium oxide

A 1.01 g of the sample of ethylmaltol (EtMa, 7.14 mmol; 2 equiv) was dissolved in 10 mL of DI H_2_O in a 50 mL round-bottom flask, with constant stirring at 90 °C (Scheme 2). A separate solution of 158.6 mg of magnesium oxide (MgO:3.93 mmol; 1.1 equiv) was taken up in 10 mL of DI H_2_O, with the addition of 172.3 mg of citric acid (CA, 0.25 equiv), constantly stirred and heated to 90 °C. The MgO/CA solution was subsequently added to the ethylmaltol solution in small increments over 5 min. Upon addition, the mixture was a translucent white color that solubilized in about 30 s; each subsequent addition was administered when the previous addition had become wholly soluble. After all additions, the reaction was noted as colorless and clear. The reaction was conducted for 1 h, whereupon the solution was noted as clear and amber/orange in color. The solution was allowed to cool to room temperature and the pH was noted as 7.80. The solution was dried *in vacuo*, at which time a tan solid was observed. The yield was found to be stoichiometric relative to ethylmaltol, and the purity was 93.9% based on ^1^H NMR. The solubility of 2 was determined to be 16.2 g/100 mL H_2_O. 2: ^1^H NMR (D_2_O, 400MHz): δ 4.79 (s, 1H), 7.99–7.98 (d, 1H, H1, *J* = 5.26Hz), 6.48–6.47 (d, 1H, H2, *J* = 5.50 Hz), 2.76–2.71 (q, 2H, H3, *J*1 = 22.62 Hz, *J*2 = 7.70 Hz), 1.19–1.15 (t, 3H, H4, *J* = 15.16Hz). EA: Theo for {[Mg(C_7_H_7_O_3_)_2_(H_2_O)_2_]·H_2_O}: C = 46.32%, H = 5.47%; Exp: C = 46.95%, H = 5.05% (Figure S1)

### 3.6 Determining Magnesium Complex Uptake in CaCo-2 Human Cells

A colorimetric magnesium uptake assay kit for use with a 96-well plate was purchased from BioVision (Milpitas, CA). Sample solutions for use with the kit were prepared in-house utilizing magnesium/calcium-free Hank’s Balanced Salt Solution (HBSS). The samples tested were magnesium chloride hexahydrate (MgCl_2_·6H_2_O), **1**, and **2**. The kit-provided standard for linearity confirmation began as a 150 nm/μL stock, as such MgCl_2_·6H_2_O, utilized as an internal standard, and was prepared at this concentration, containing 17.93 mM Mg^2+^. Both **1** and **2** were prepared to contain the same amount of Mg^2+^ to evaluate magnesium uptake in a relative fashion. DMEM was removed from the plated cells and cells were subsequently washed three times with HBSS in 100 μL volume. MgCl_2_, **1**, and **2** were administered at 150 μL/well as triplicate independent dilutions. Cells were treated for 1–2 h at 37 °C and 5% CO_2_. After incubating, the sample volume was removed from each well and the cells were again washed three times with HBSS. Cells were lysed utilizing 200 μL of kit assay buffer, the post-lysis volume was collected, and each sample was centrifuged at 14 000*g* for 10 min. The resulting supernatants were replated in the same order in 50 μL volume. Fifty μL of the kit-provided enzyme/buffer/developer mix was added to each well with a multichannel micropipette, and the plate was allowed to incubate for 40 min at 37 °C. Some wells were left blank for required background subtraction. The kit-provided standard was diluted to 0, 3, 6, 9, 12, and 15 nmol/μL in DI H_2_O and administered and developed in the same volumes as MgCl_2_·6H_2_O, **1**, and **2** and was used only to determine kit linearity (see Supporting Information (Figure S26)). Each well was analyzed for endpoint value over nine full plate scans with triplet scans/well/plate scan (a total of 27 scans per well) and the reported value of each well was the average value of these scans after background subtraction. All samples were analyzed in triplicate. Data were collected at 40 min. Raw data was reduced and plotted as absorbance against the magnesium concentration of each well. All assays were repeated in triplicate (SEM, MgCl_2_ = ±0.0006, **1** = ±0.001, **2** = ±0.001; Upper 95% C.I., MgCl_2_ = 0.004, **1** = 0.008, **2** = 0.011; Lower 95% C.I. = MgCl_2_ = 0.001, **1** = 0.001, **2** = 0.001).

## Supporting Information Available

Synthesis of **1** and **2**, ESMS of **1** and **2**, ^1^H NMR of maltol/ethylmaltol and **1**/**2**, ^13^C NMR of maltol/ethylmaltol and **1**/**2**, HSQC/HMBC of maltol/ethylmaltol and **1**/**2**, TGA/DSC of maltol/ethylmaltol and **1**/**2**, EA of recovered maltol/ethylmaltol and synthesized **1**/**2**, FT-IR of maltol/ethylmaltol and **1**/**2** (PDF)

## Abbreviations

**1**: magnesium maltol
**2**: magnesium ethylmaltol
CaCo-2: colorectal carcinoma cells differentiated into human intestinal epithelial cells
DMEM: Dulbecco’s Modified Eagle Medium
HBSS: Hank’s Balanced Salt Solution
MgCl_2_: magnesium chloride
MgO: magnesium oxide
ESMS: electrospray Mass Spectrometry
NMR: nuclear magnetic resonance
HSQC: heteronuclear single quantum coherence
HMBC: heteronuclear multiple bond correlation
FT-IR: Fourier transform infrared radiation
TGA: thermogravimetric analysis
DSC: differential scanning calorimetry
EA: elemental analysis
