# Supplementary material for: Synthesis, Characterization, and Cellular Uptake of Magnesium Maltol and Ethylmaltol Complexes
Source: ACS Omega. 2021 Oct 28;6(44):29713–23. doi: 10.1021/acsomega.1c04104 (PMC8587132; doi:10.1021/acsomega.1c04104)
Supplement: Supplementary file 1 — ao1c04104_si_001.pdf [file ao1c04104_si_001.pdf]

Supplemental Information

Synthesis, Characterization and Cellular Uptake of Magnesium Maltol and Ethylmaltol Complexes

Derek R. Case,[†] Ren Gonzalez,[‡] Jon Zubieta,[†] Robert P. Doyle[†,*]

[†]111 College Place, Department of Chemistry, Syracuse University, Syracuse, New York, 13244, USA

[‡] Balchem Corporation, 52 Sunrise Park Road, New Hampton, NY 10958, USA

[*] Correspondence: rpdoyle@syr.edu

Table of Contents

Table of Contents……………………………………………………………………………..….Pages S1-2

Elemental Analysis Values for **1** and **2** **(S1)**……………………..………………………………..Page S3

Full ^{1}H NMR of **1** **(S2)**…………………………………………………..…………………......Page S4

Full ^{1}H NMR of **2** **(S3)**……..…………………………………………………………………….…...Page S5

^{1}H NMR Overlay of Citric Acid, Magnesium Citrate, **1** and **2** **(S4)**..……………..………………...Page S6

FT-IR Overlay of Maltol/**1** **(S5)**………………………………………………………………........Page S7

FT-IR Overlay of Ethylmaltol/**2** **(S6)**…………………………………………………………….......Page S8

Full ^{1}H NMR of Maltol **(S7)**……………………………………………………………………..…….Page S9

Full ^{1}H NMR Overlay of Maltol and **1** **(S8)**……………………………………………..….…..…...Page S10

^{1}H NMR Overlay of Maltol and **1** in the Aliphatic Region **(S9)**……………………...…...…...….Page S11

^{1}H NMR Overlay of Maltol and **1** in the Aromatic Region **(S10)**…………………………….……...Page S12

Full ^{13}C NMR of Maltol **(S11)**……………………………………………………………….…….Page S13

Full ^{1}H-^{13}C HSQC of Maltol **(S12)**…………………………………………………………………..Page S14

Full ^{1}H-^{13}C HMBC of Maltol **(S13)**……………………………………………………..…....Page S15

Full ^{1}H-^{13}C HSQC of **1** **(S14)**………………………………………………………..…..…..Page S16

Full ^{1}H-^{13}C HMBC of **1** **(S15)**………………………………………………………..…..….....Page S17

Full ^{1}H NMR of Ethylmaltol **(S16)**………………………………………………………......Page S18

^{1}H NMR Overlay of Ethylmaltol and **2** (Full) **(S17)**……………..……………………...……Page S19

^{1}H NMR Overlay of Ethylmaltol and **2** (Aromatic) **(S18)**…………………………...…………….Page S20

^{1}H NMR Overlay of Ethylmaltol and **2** (Aliphatic) **(S19)**……………………………...……..….Page S21

Full ^{13}C NMR of **2** **(S20)**……………………………………………………………………….…..Page S22

Full ^{1}H-^{13}C HSQC of Ethylmaltol **(S21)**…………………………………………………….………..Page S23

Full ^{1}H-^{13}C HMBC of Ethylmaltol **(S22)**……………………………………………………….……..Page S24
Full ^{1}H-^{13}C HSQC of **2 (S23)**……………………………………………..……………………..….Page S25
Full ^{1}H-^{13}C HMBC of **2 (S24)**……………………………………………………..……………..…Page S26
Full ^{13}C NMR of Ethylmaltol **(S25)**……………………………………………………..…………......Page S27
Linear Regression of Cell Uptake Kit **(S26)**……………………………………………………………Page S28

Analytical Report
Report Number: 181975
Report Status: *Final*

Derek Case
Syracuse University
111 College Place
O-006 CST Bldg
Syracuse, NY 13244

Sample: Mg(Malt)2 - 1.2MgO

C	H
43.31 %	4.39 %
43.34 %	4.59 %

Elemental Analysis Values

1	Carbon %	Hydrogen %
Calculated Values	43.86%	4.91%
Sample 1	43.31%	4.39%
Sample 2	43.34%	4.59%
Average Mass %	43.33%	4.49%
Average Error	-0.53%	-0.42%

Analytical Report
Report Number: 175596
Report Status: *Final*

Derek Case
Syracuse University
111 College Place
O-006 CST Bldg
Syracuse, NY 13244

Sample: MgEtma

C	H
46.89 %	5.13 %
47.00 %	4.97 %

Elemental Analysis Values

2	Carbon %	Hydrogen %
Calculated Values	46.89%	5.62%
Sample 1	46.89%	5.13%
Sample 2	47.00%	4.97%
Average Mass %	46.95%	5.05%
Average %Error	0.05%	-0.57%

Figure S1. EA Values for **1** and **2**.

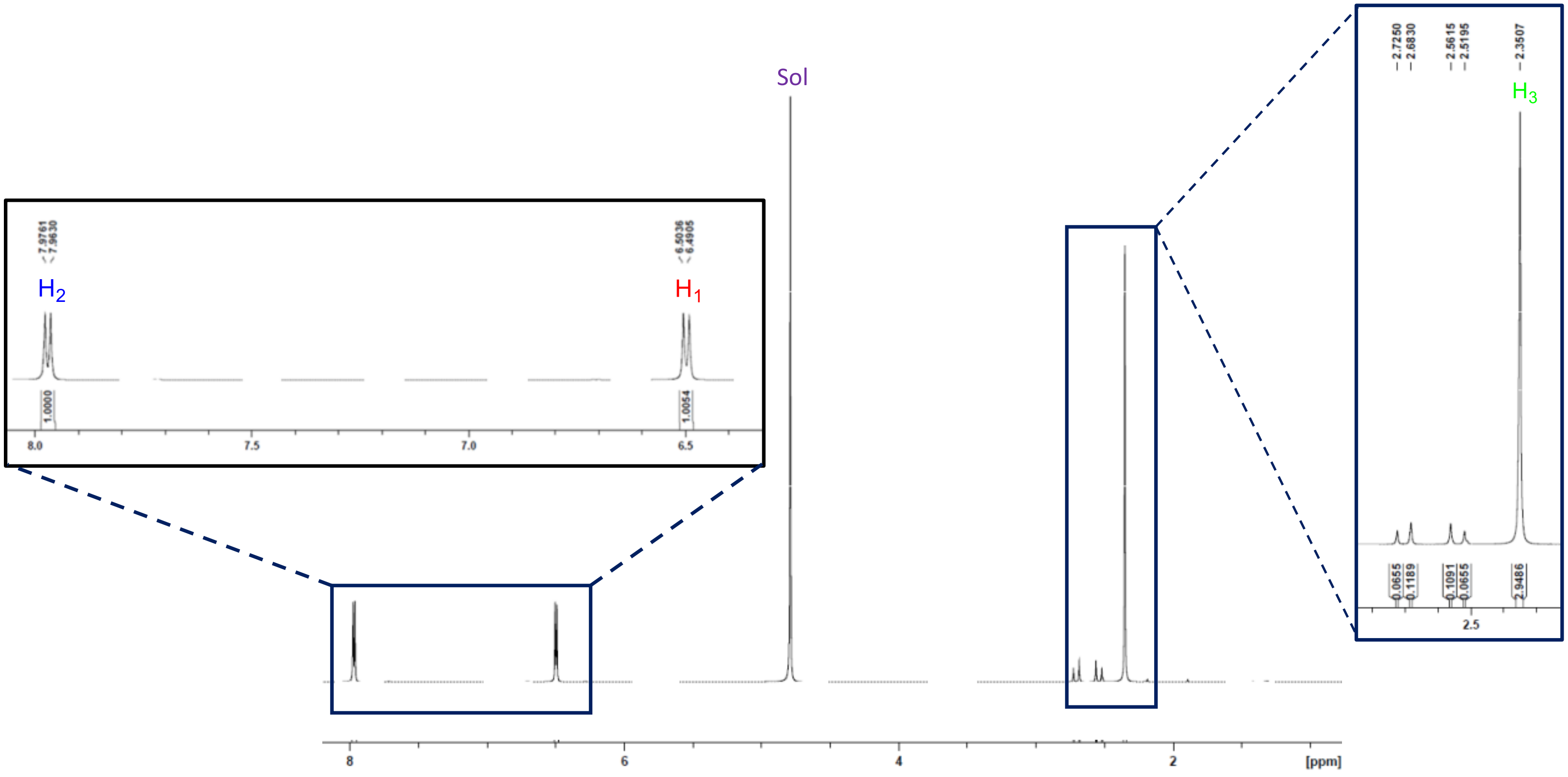

Figure S2. Full ^{1}H NMR spectrum of **1** with both the aromatic and aliphatic regions enlarged (**inset right**) – shows the presence of magnesium citrate at 2.72, 2.68, 2.56, and 2.51ppm, respectively.

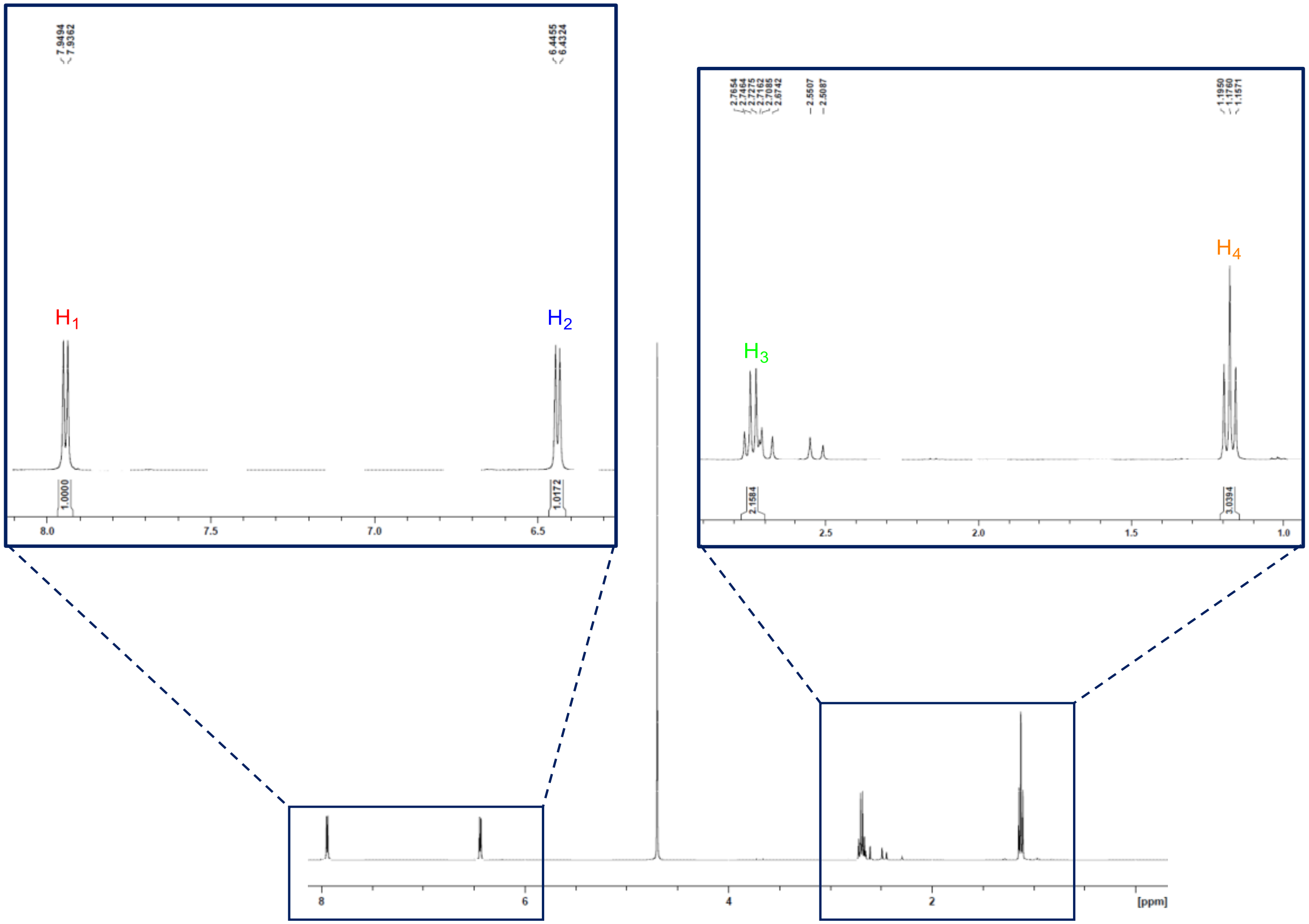

Figure S3. Full ^{1}H NMR of **2** with both the aromatic (**inset left**) and aliphatic (**inset right**) regions enlarged showing magnesium citrate at 2.70, 2.67, 2.55, and 2.50ppm, respectively.

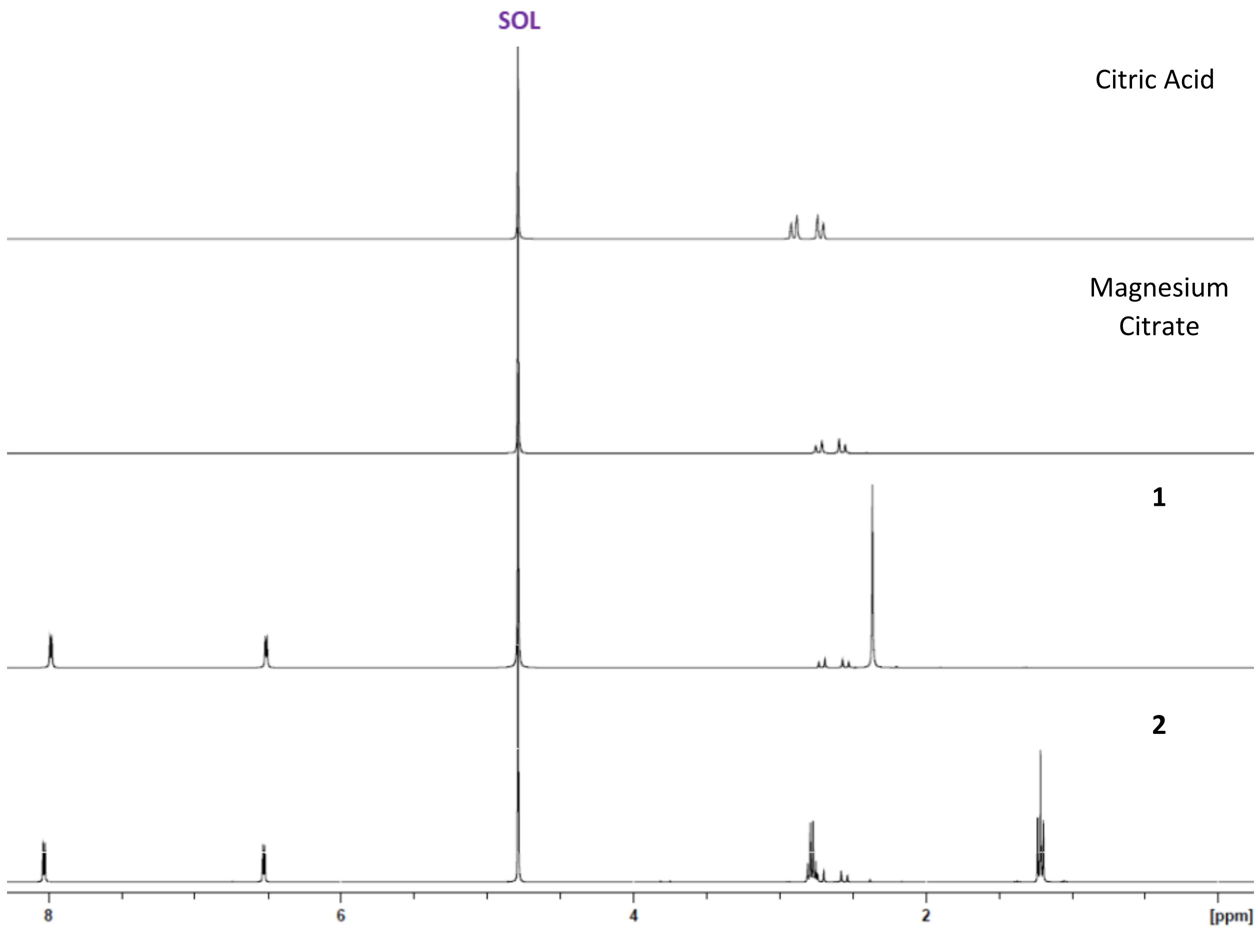

Figure S4. ^{1}H NMR confirming the presence of *in situ* formed magnesium citrate in both **1** and **2.**

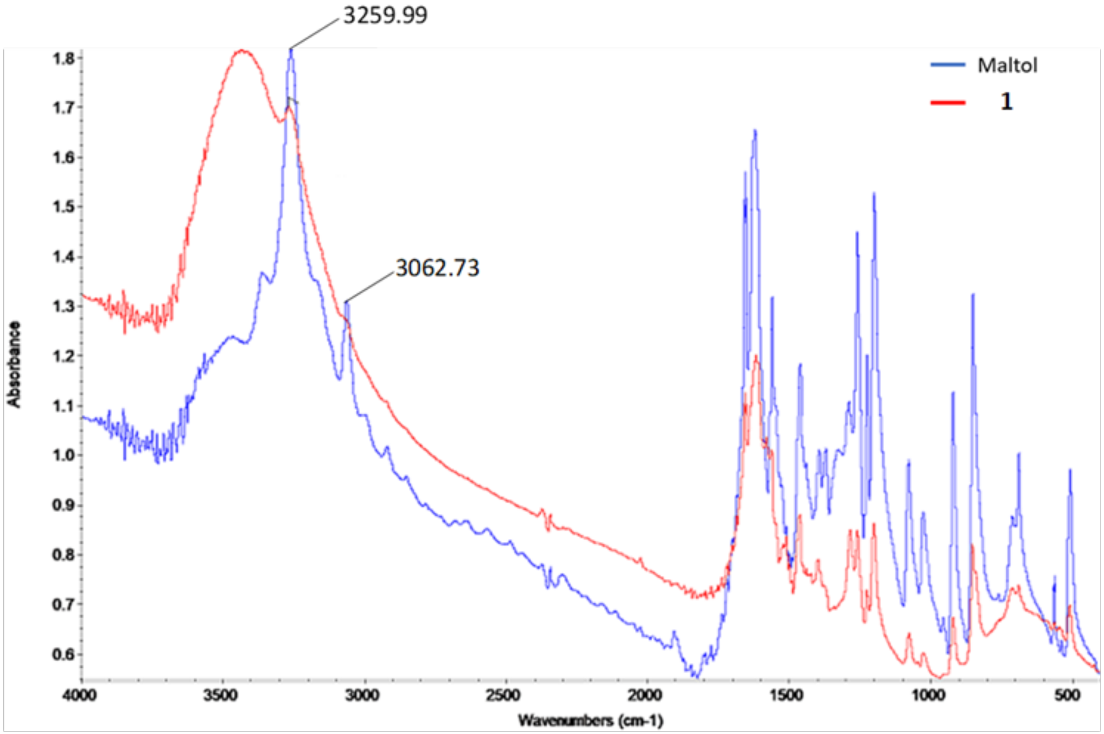

Region of Interest	Functional Group	Maltol	MgMalt
$1650 cm^{-1}$ - $1750 cm^{-1}$	C=O	$1654.93 cm^{-1}$	$1654.93 cm^{-1}$
$3000 cm^{-1}$ - $3500 cm^{-1}$	v(OH)	$3062.73 cm^{-1}$	--
$3000 cm^{-1}$ - $3500 cm^{-1}$	v(OH)	$3259.99 cm^{-1}$	$3259.99 cm^{-1}$

Figure S5. FT-IR overlay of maltol and **1** the near-complete quenching of the band attributed to the alcohol**.**

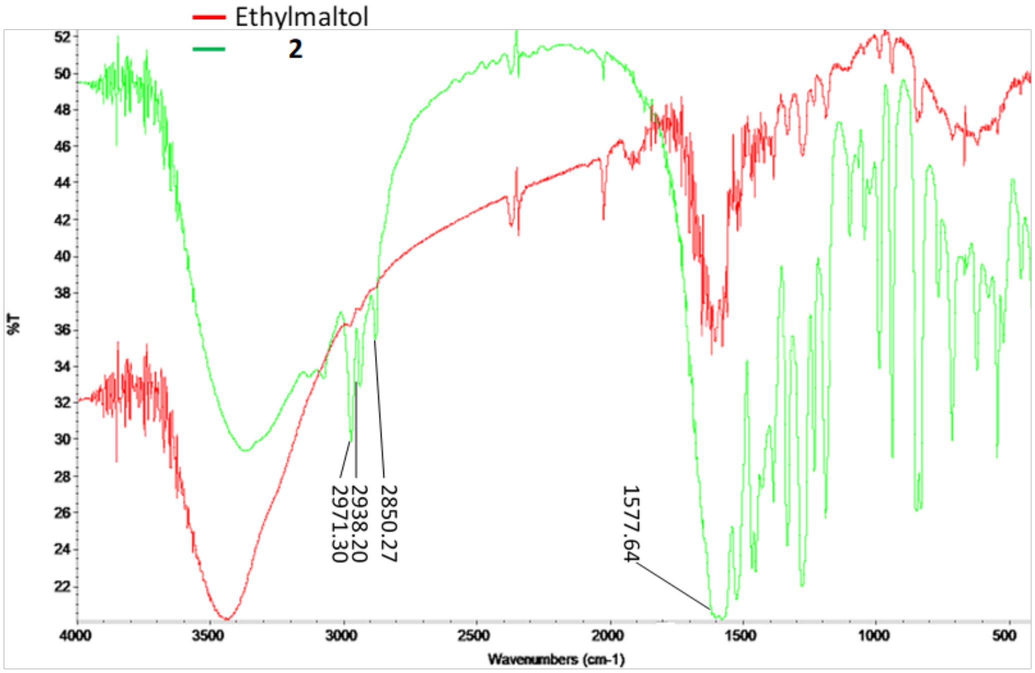

Region of Interest	Functional Group	Ethylmaltol	2
$1650cm^{-1}$ - $1750cm^{-1}$	C=O	$1577.64cm^{-1}$	$1577.64cm^{-1}$
$2900cm^{-1}$ - $3100cm^{-1}$	ν(OH)	$2850.27cm^{-1}$	--
$3000cm^{-1}$ - $3500cm^{-1}$	ν(OH)	$2938.20cm^{-1}$	--
$3000cm^{-1}$ - $3500cm^{-1}$	ν(OH)	$2971.30cm^{-1}$	--

Figure S6. FT-IR overlay of ethylmaltol and **2** showing the quenching of the bands attributed to the alcohol**.**

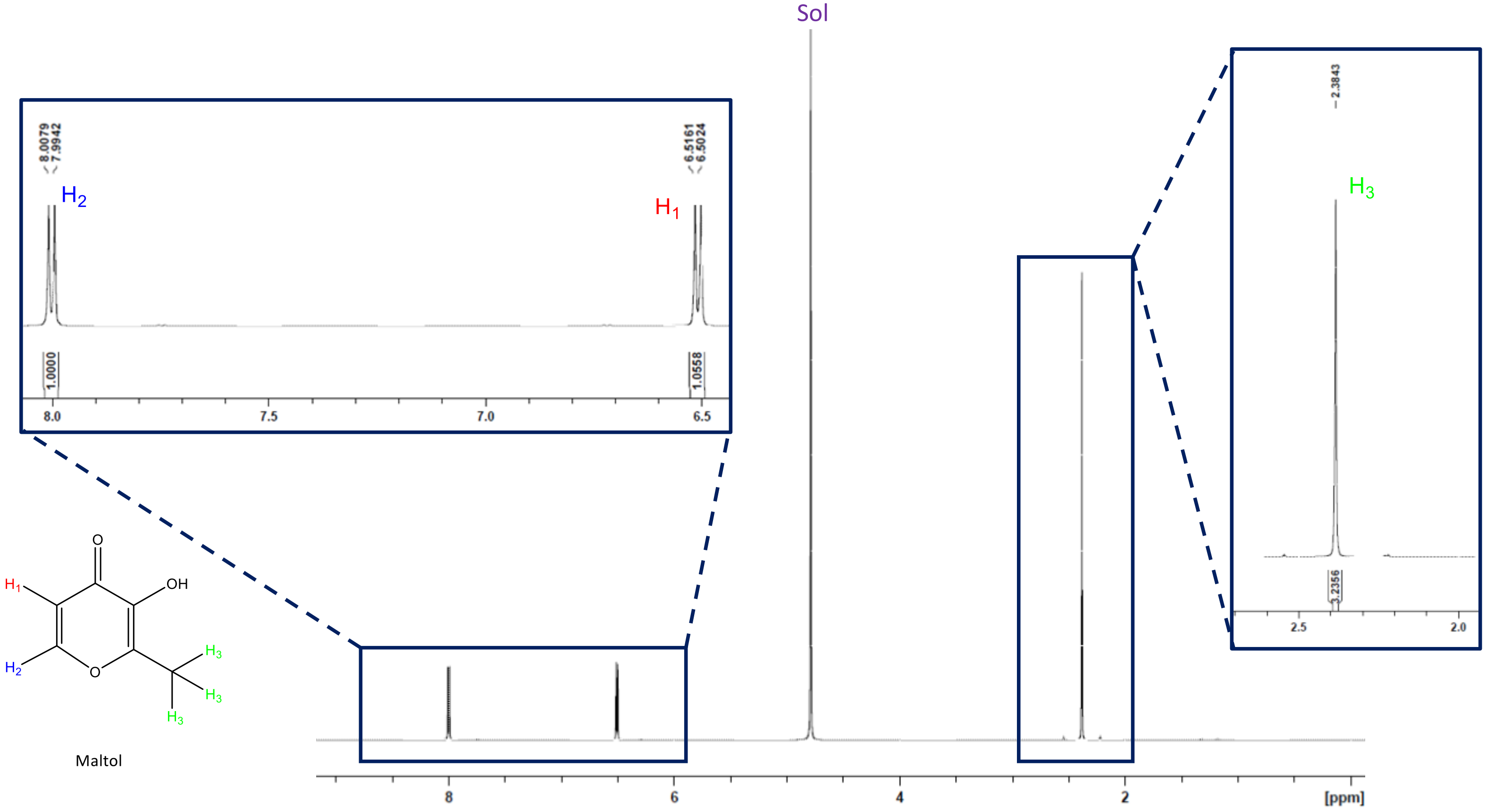

Figure S7. Full ^{1}H NMR of maltol with both the aromatic (**inset left**) and aliphatic (**inset right**) regions enlarged.

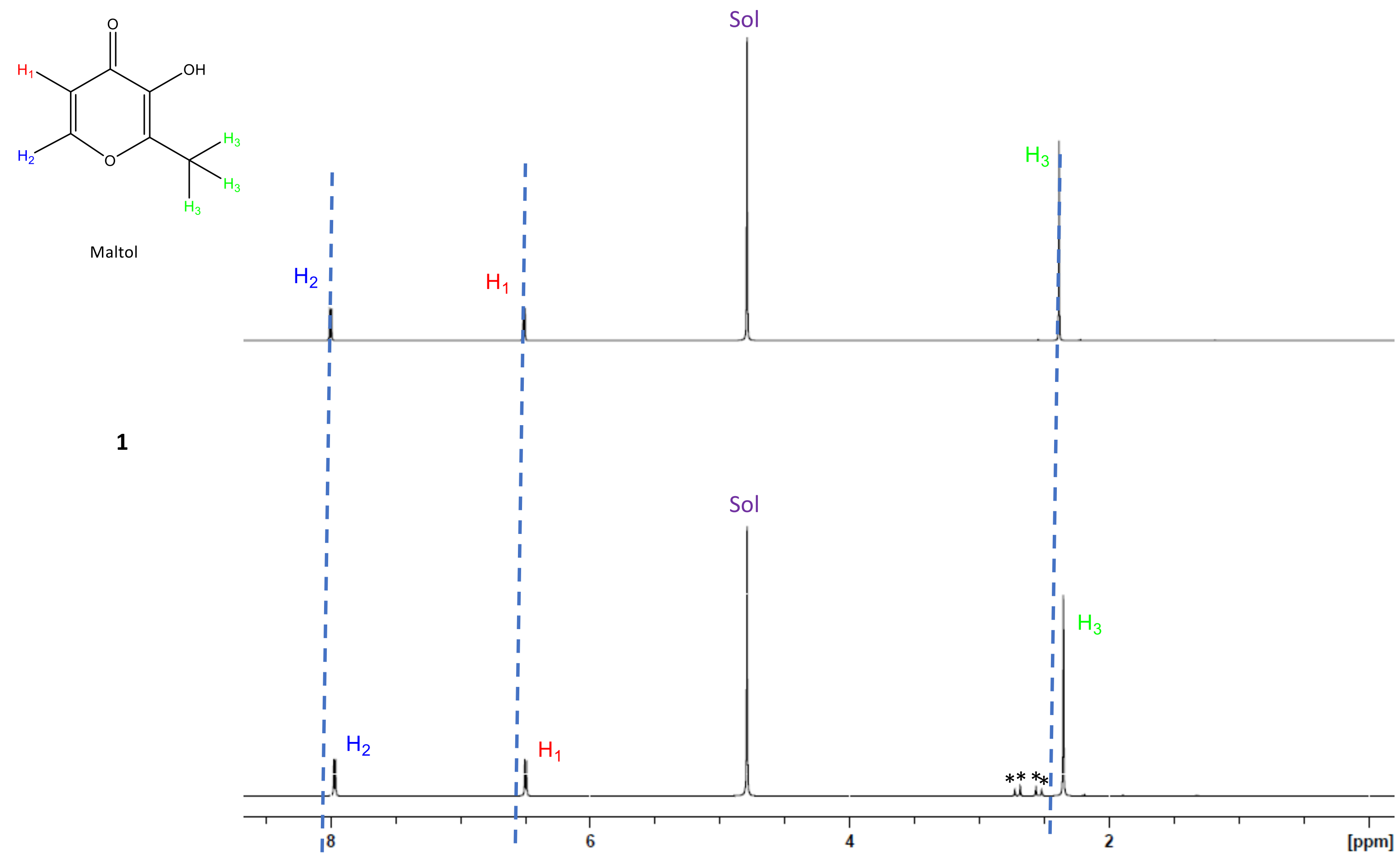

Figure S8. Full ^{1}H NMR overlay of maltol and **1.**

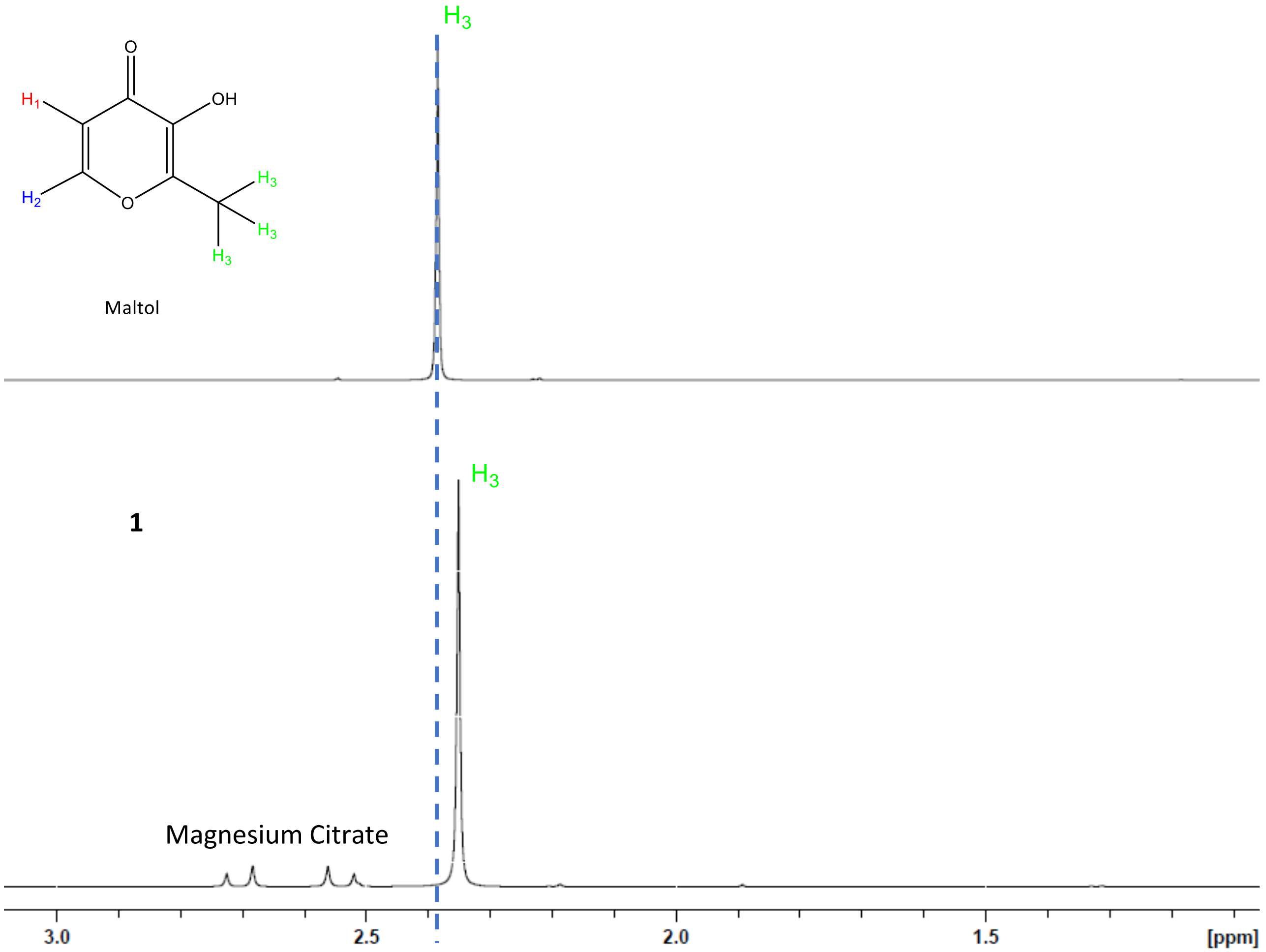

Figure S9. ^{1}H NMR overlay of maltol and **1** zoomed in the aliphatic region.

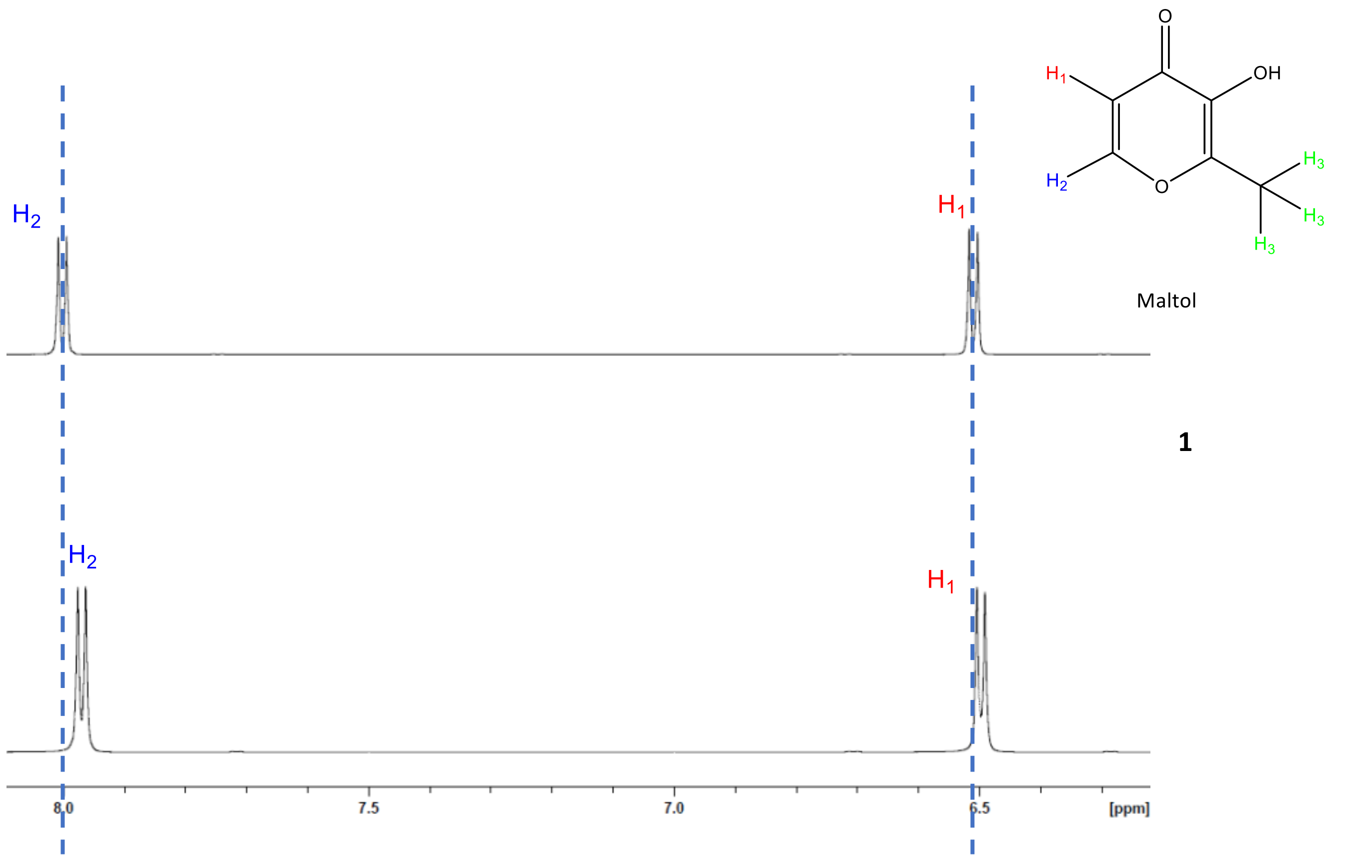

Figure S10. ^{1}H NMR overlay of maltol and **1** zoomed in the aromatic region.

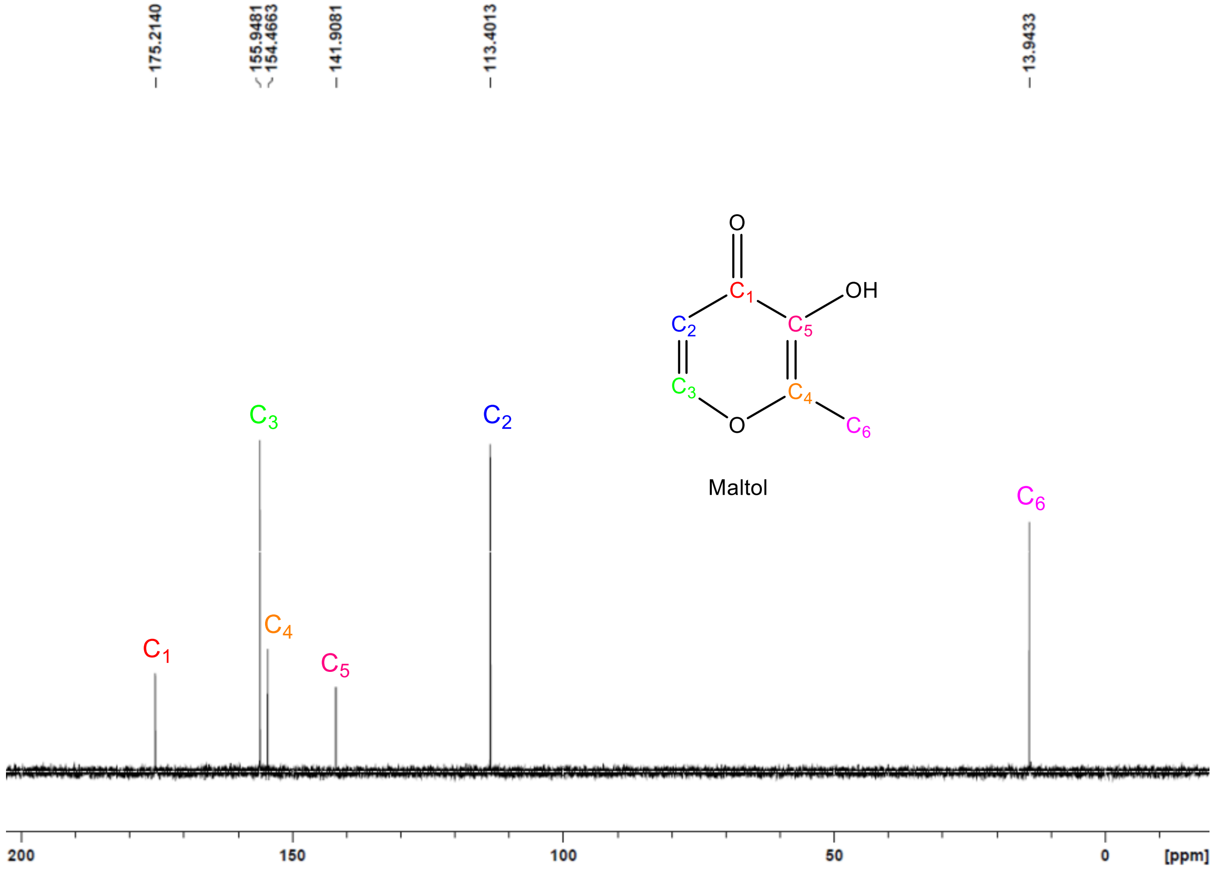

Figure S11. Full ^{13}C NMR of maltol.

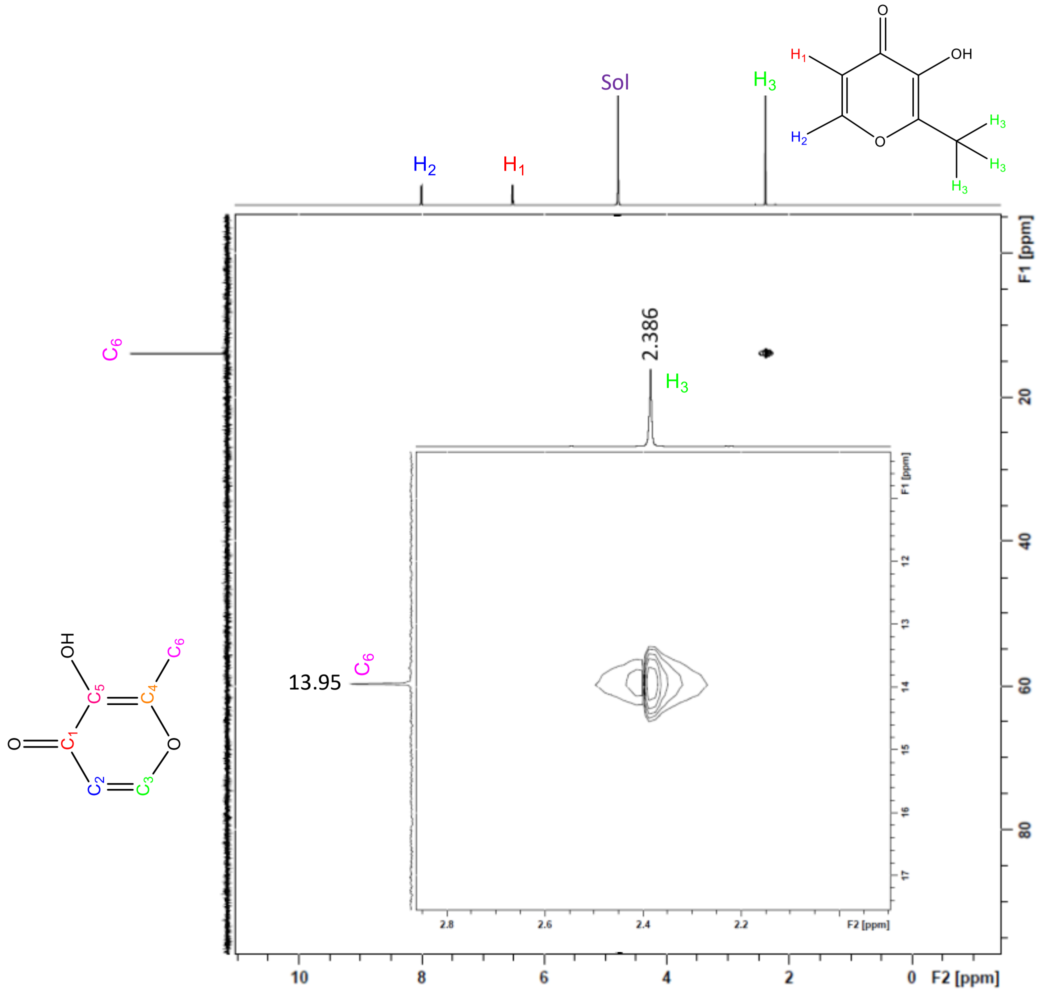

HSQC 2D NMR Peaks		
Peak	Proton Shift	Carbon Shift
1	2.386	13.95

Figure S12. Full ^{1}H-^{13}C HSQC NMR of maltol.

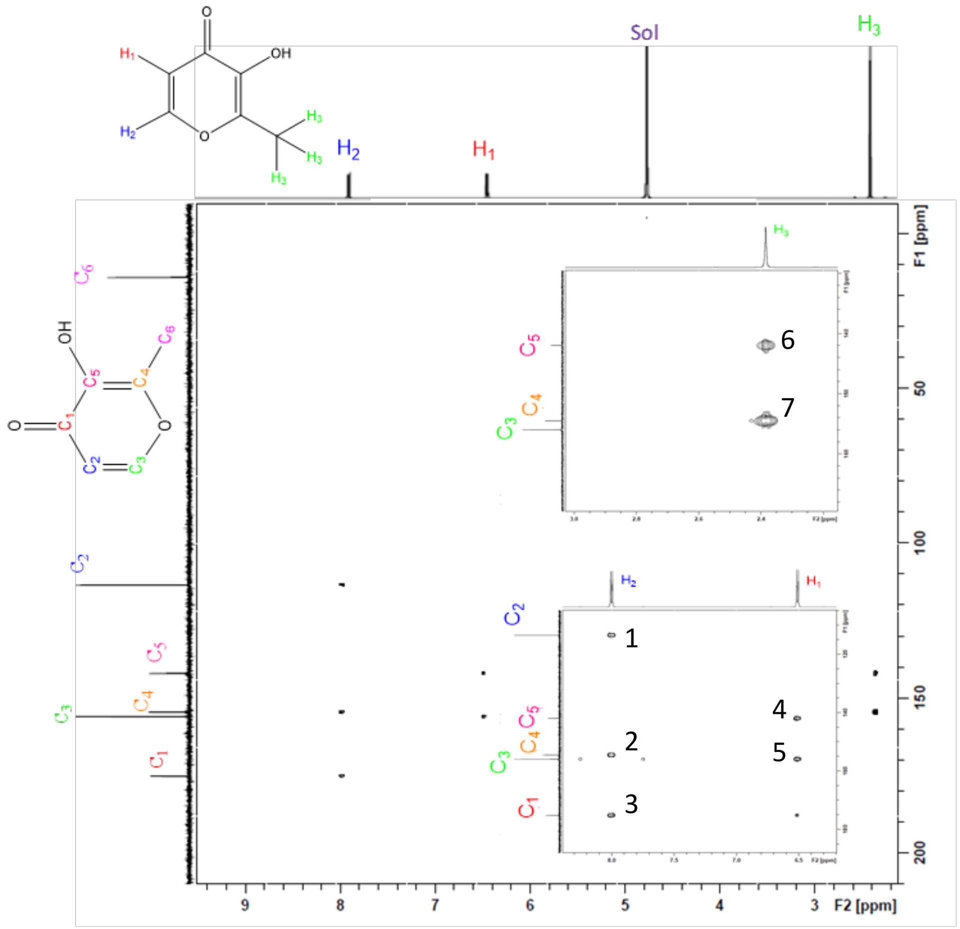

HMBC 2D NMR Peaks		
Peak	Proton Shift	Carbon Shift
1	7.999	113.40
2	7.999	154.50
3	7.999	175.20
4	6.513	142.00
5	6.513	156.00
6	2.386	142.00
7	2.386	154.50

Figure S13. Full ^{1}H-^{13}C HMBC NMR of maltol.

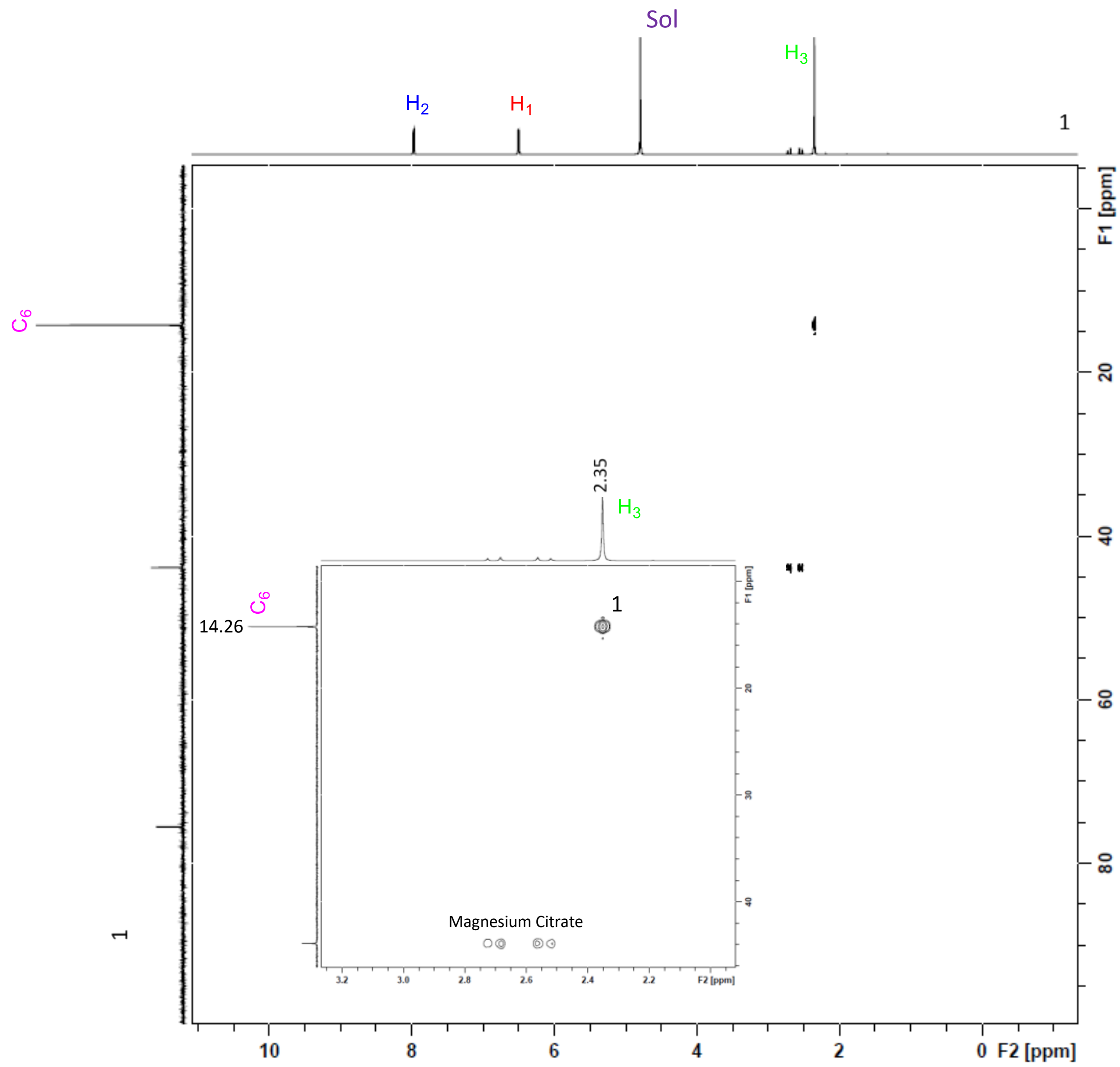

Figure S14. Full ^{1}H -^{13}C HSQC of **1** with the presence of magnesium citrate observed independent of any correspondence to peaks attributed to magnesium maltol.

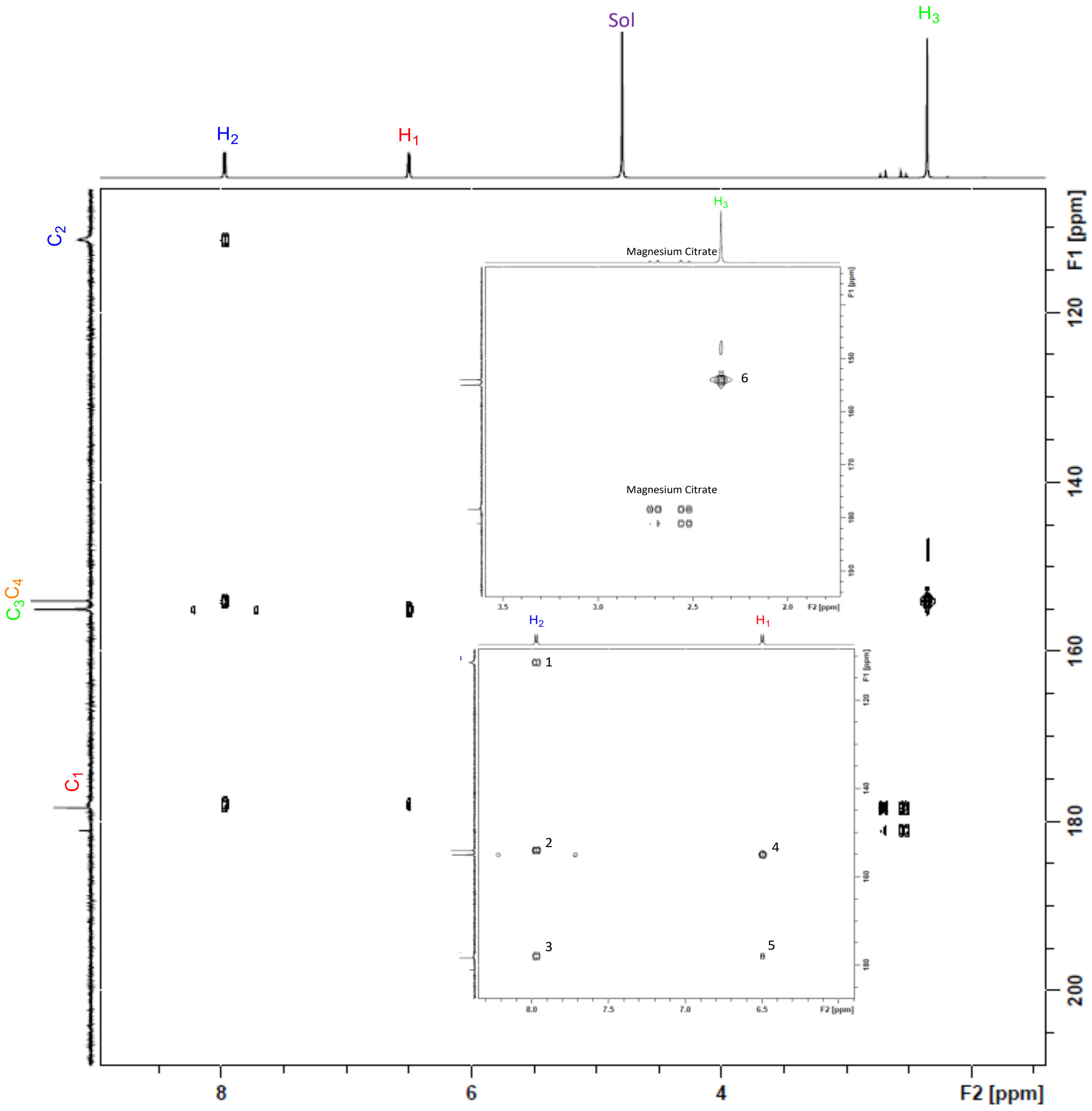

HMBC 2D NMR Peaks		
Peak	Proton Shift	Carbon Shift
1	7.970	111.34
2	7.970	154.06
3	7.970	178.32
4	6.487	155.89
5	6.487	178.05
6	2.353	154.07

Figure S15. Full ^{1}H-^{13}C HMBC of **1.**

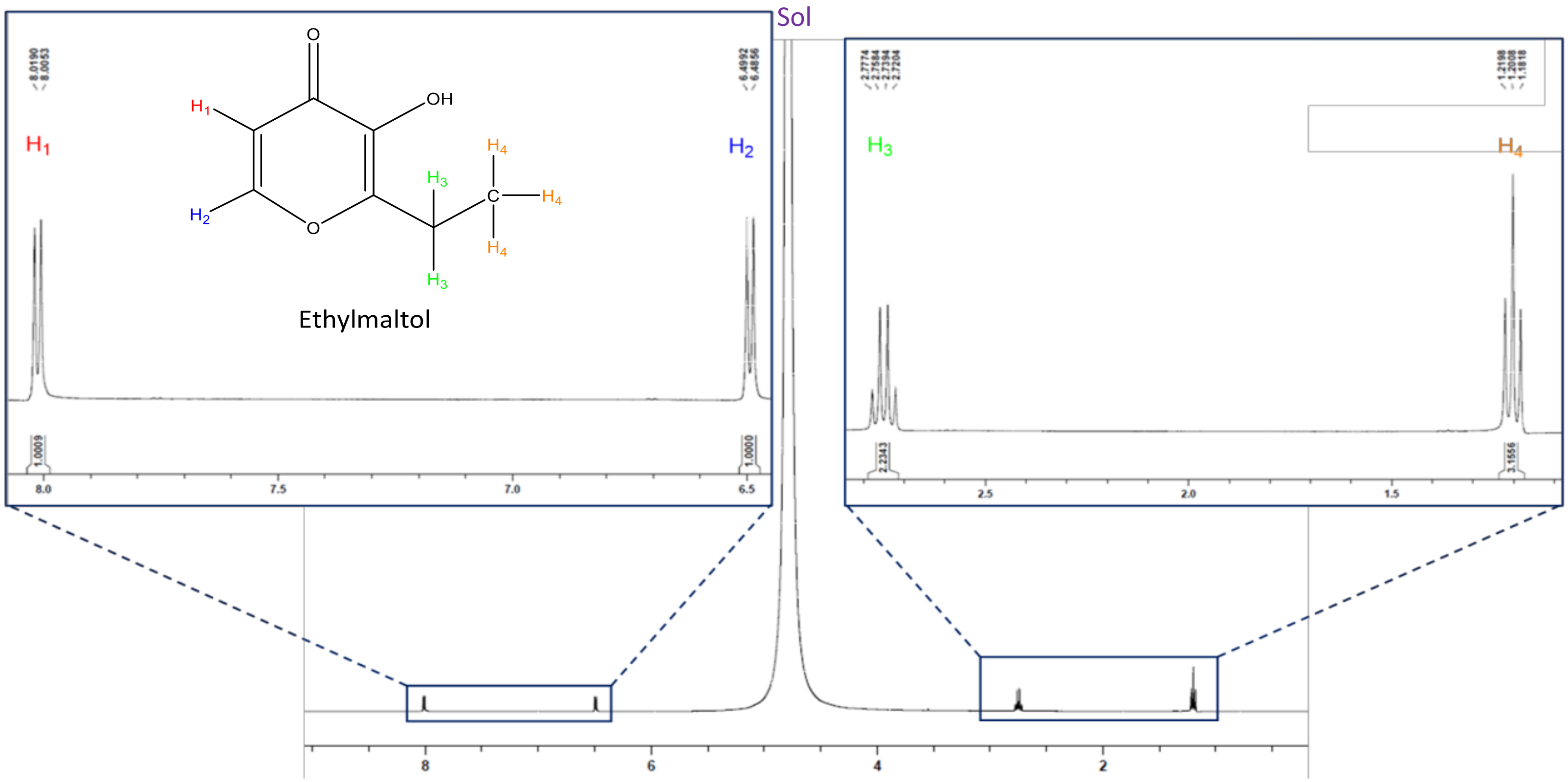

Figure S16. Full ^{1}H NMR of ethylmaltol with both the aromatic (**inset left**) and aliphatic (**inset right**) regions enlarged.

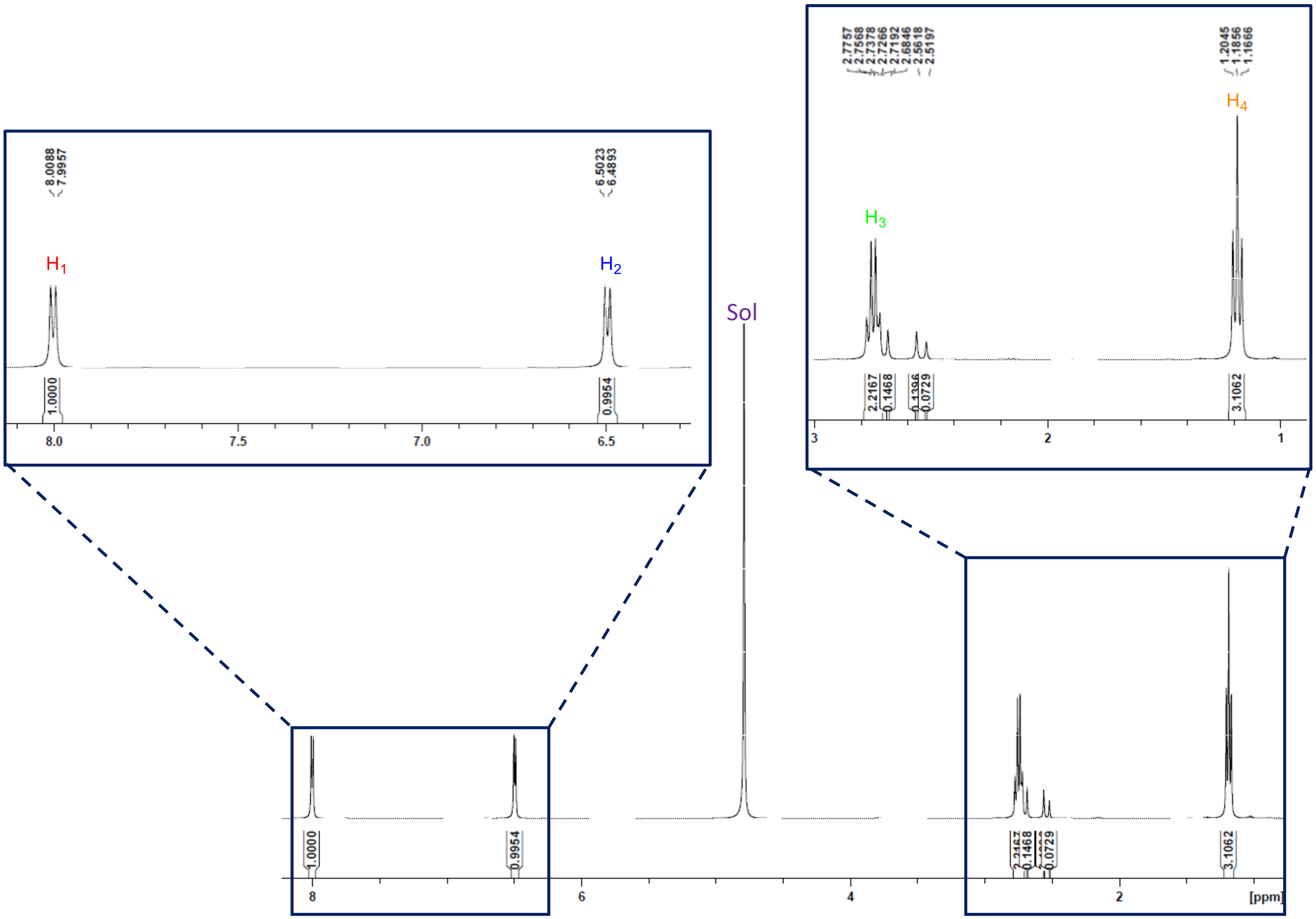

Figure S17. Full ^{1}H NMR of **2** with both the aromatic (**inset left**) and aliphatic (**inset right**) regions enlarged showing magnesium citrate at 2.71, 2.68, 2.56, and 2.51ppm, respectively.

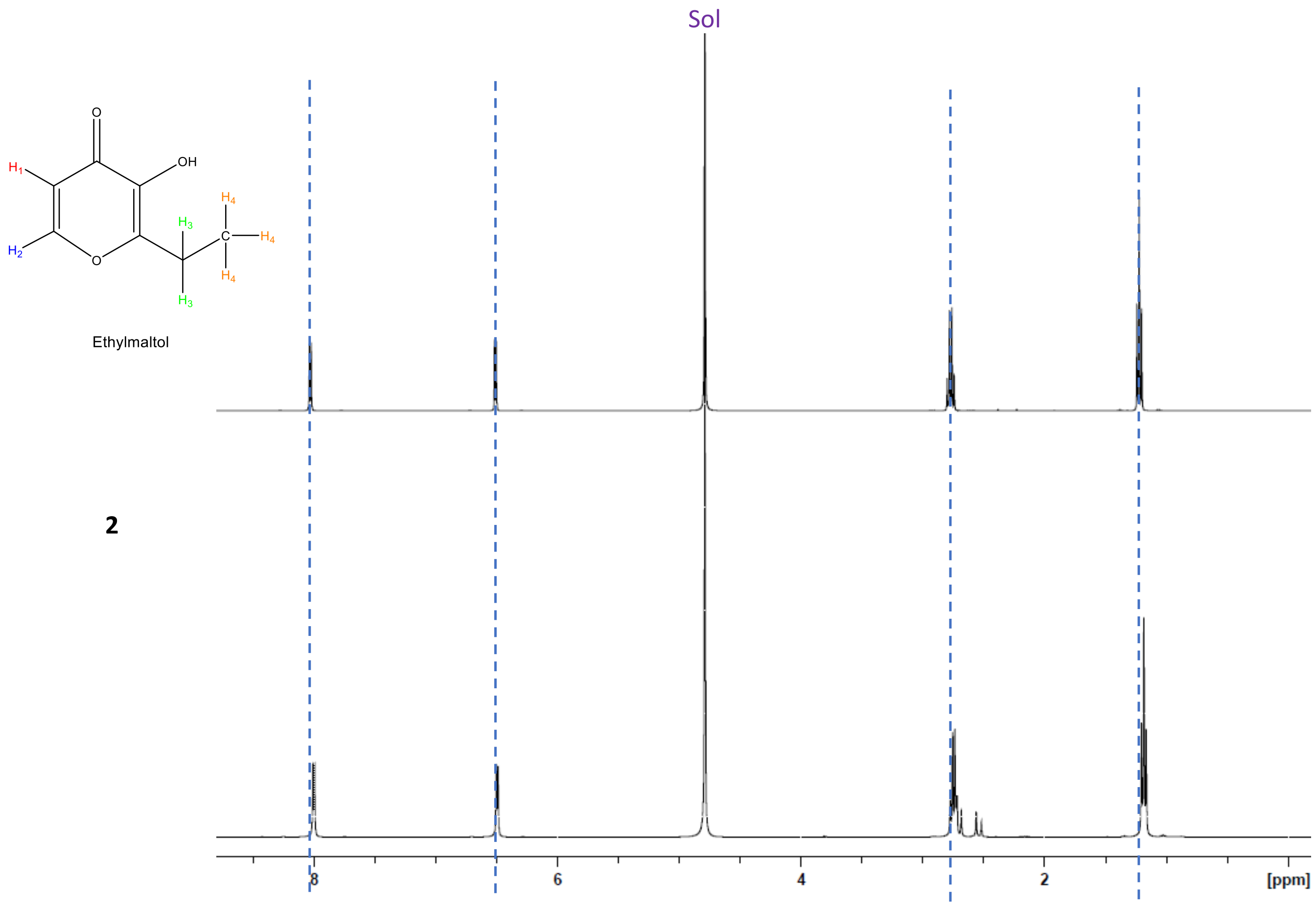

Figure S18. Full ^{1}H NMR overlay of ethylmaltol and **2.**

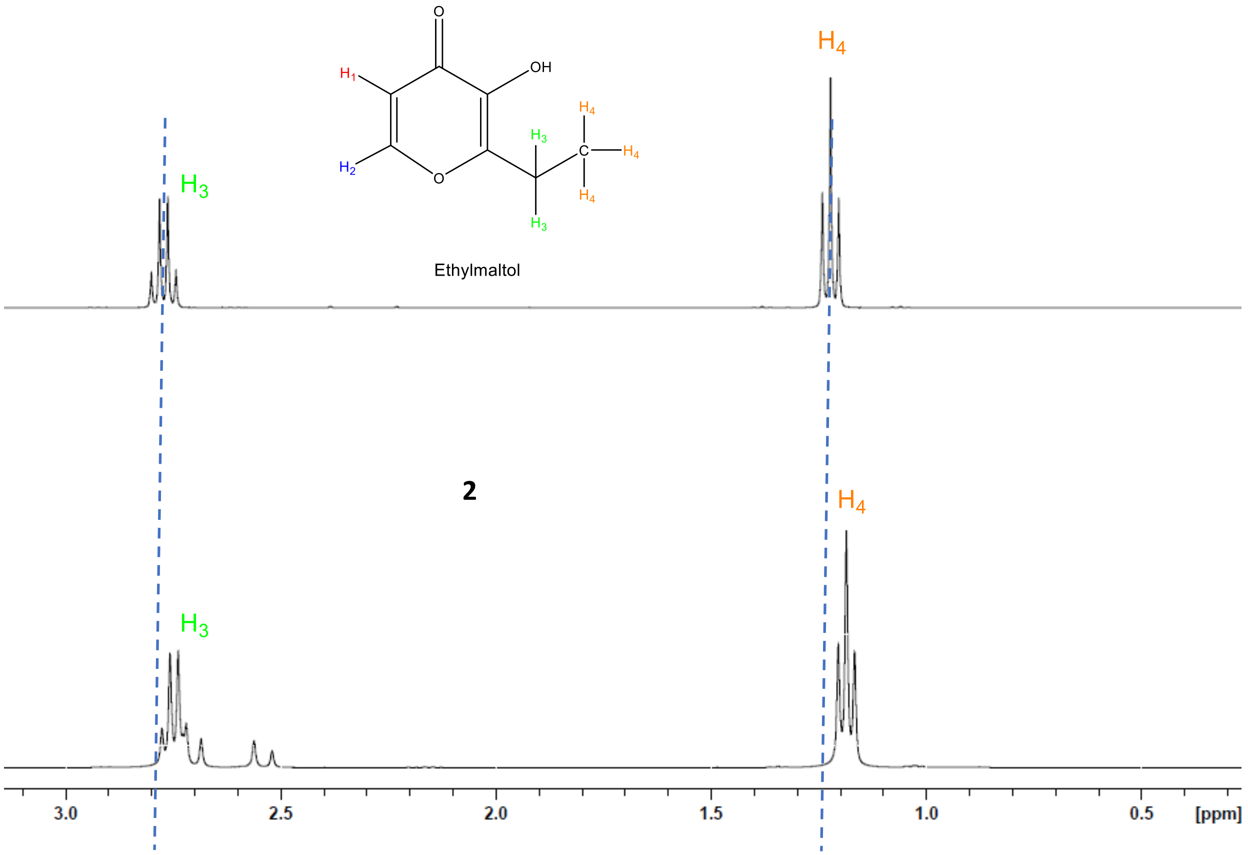

Figure S19. ^{1}H NMR overlay of ethylmaltol and **2** zoomed in the aliphatic region.

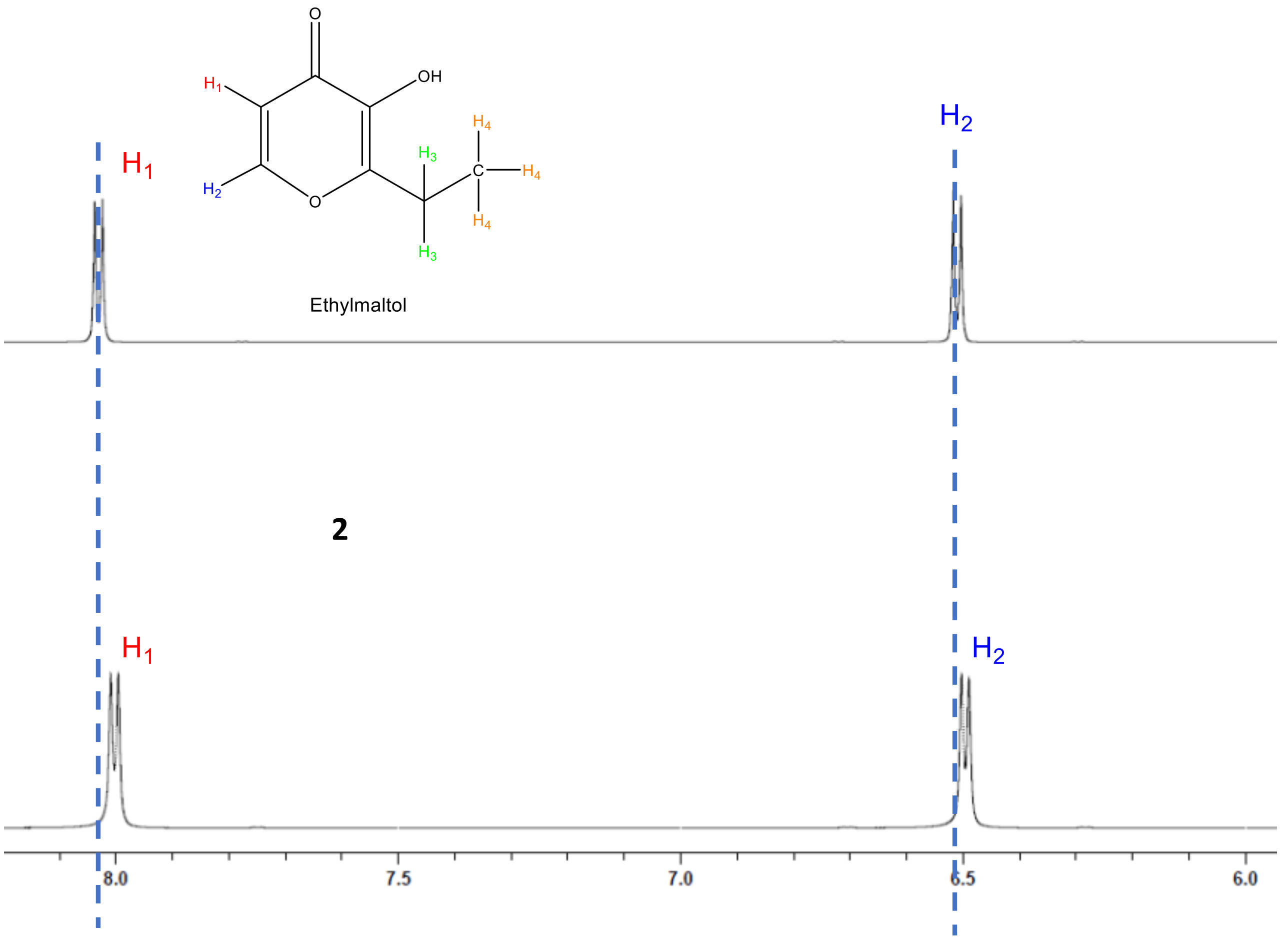

Figure S20. ^{1}H NMR overlay of ethylmaltol and **2** zoomed in the aromatic region.

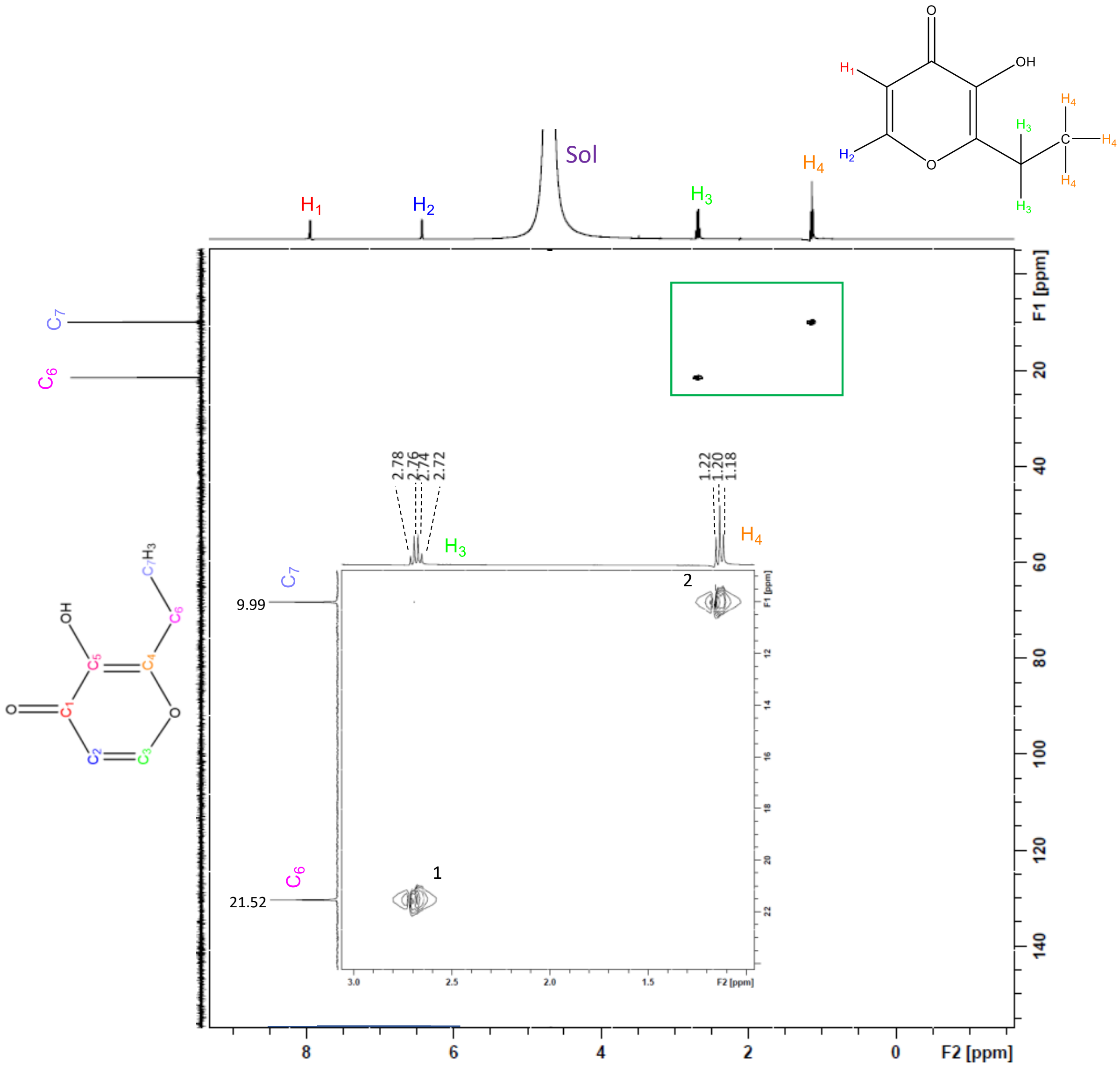

HSQC 2D NMR Peaks		
Peak	Proton Shift	Carbon Shift
1	2.78	21.52
	2.76	21.52
	2.74	21.52
	2.72	21.52
2	1.22	9.99
	1.20	9.99
	1.18	9.99

Figure S21. Full ^{1}H-^{13}C HSQC of ethylmaltol.

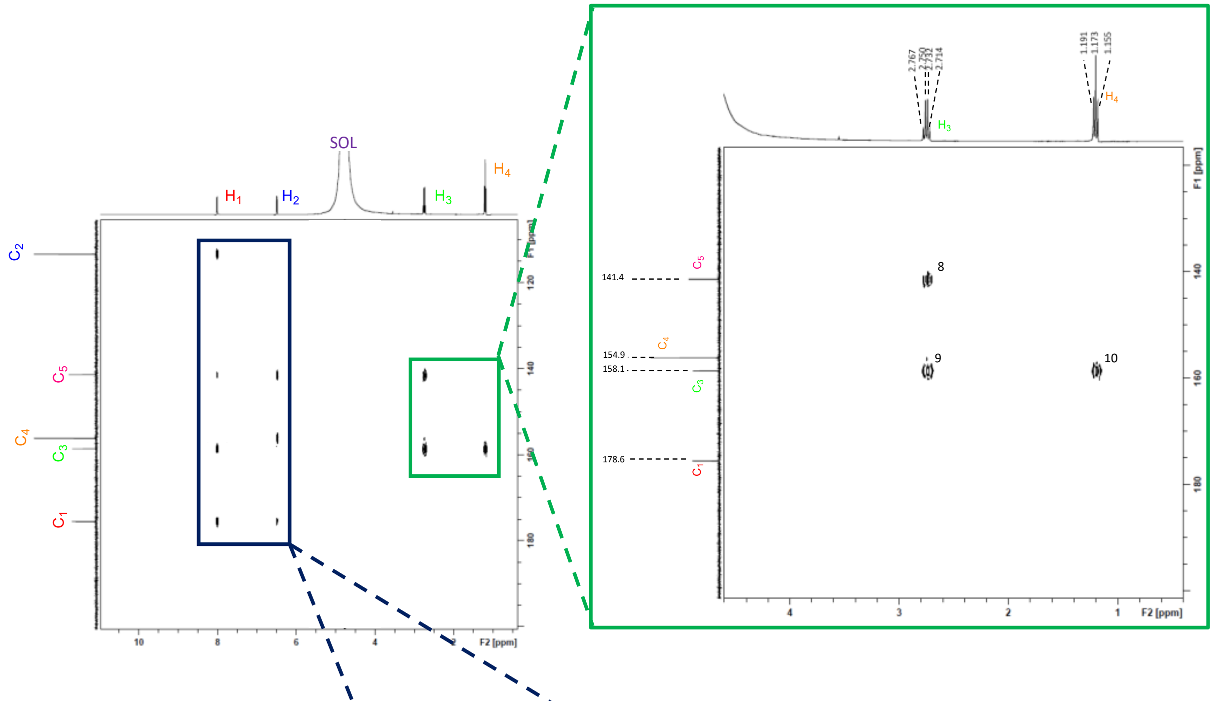

HMBC 2D NMR Peaks		
Peak	Proton Shift	Carbon Shift
1	7.99	111.20
2	7.99	141.40
3	7.99	158.10
4	7.99	178.6
5	6.48	141.4
6	6.48	154.9
7	6.48	178.6
8	2.77	141.4
9	2.77	158.3
10	1.19	158.3

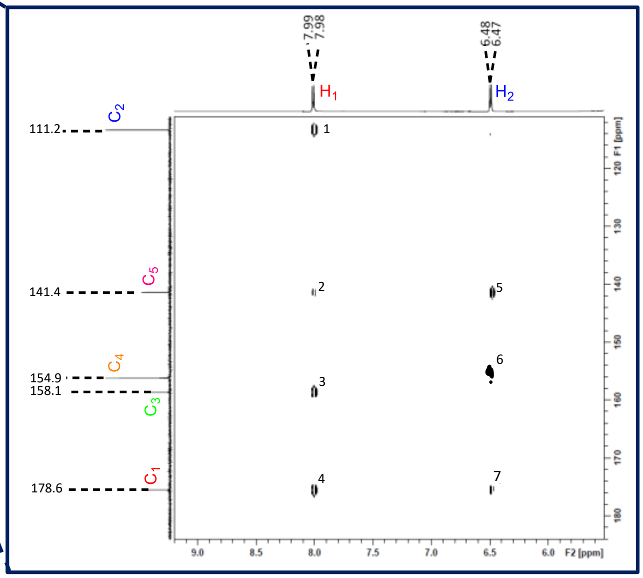

Figure S22. Full ^{1}H-^{13}C HMBC NMR of ethylmaltol.

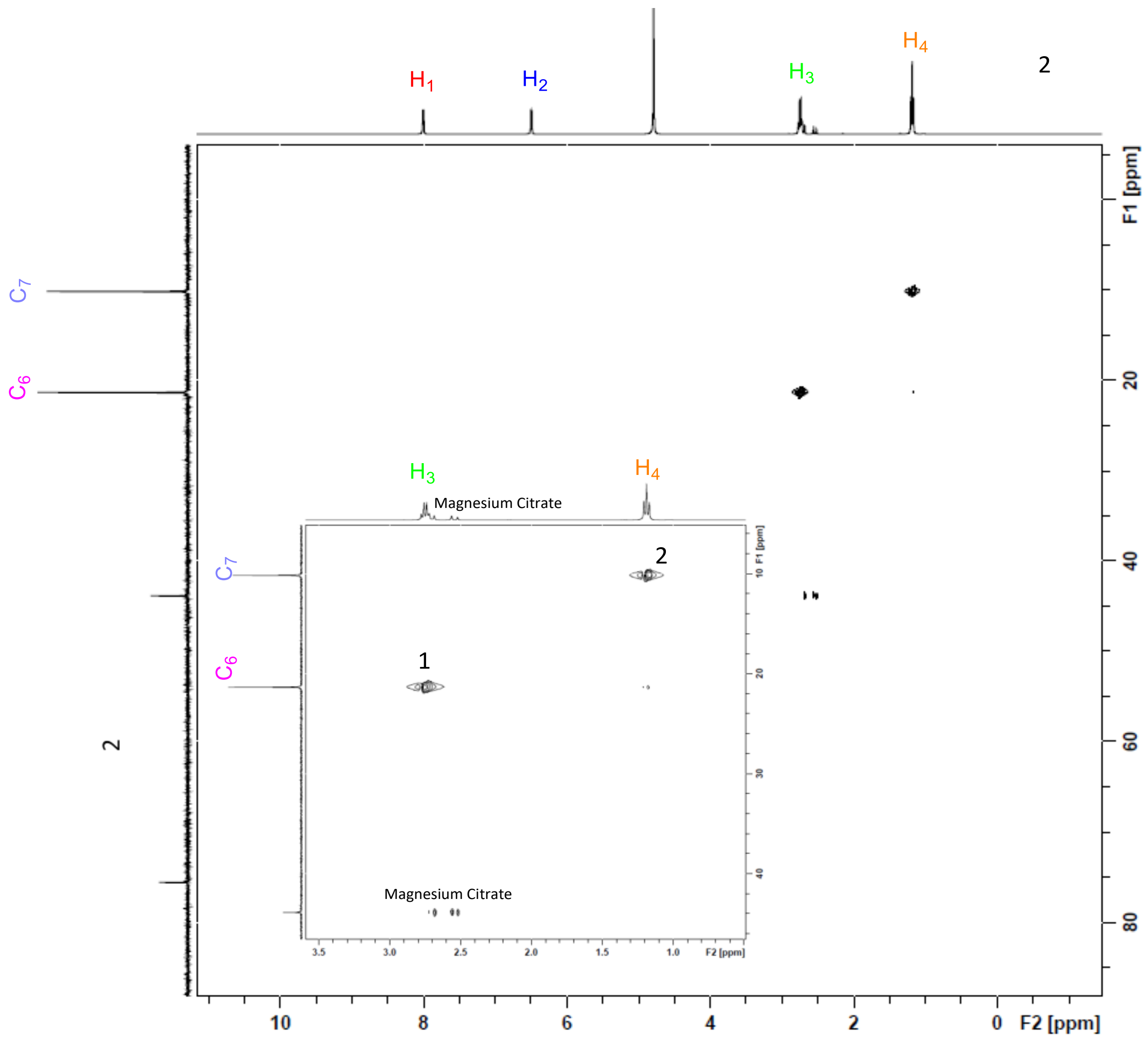

HSQC 2D NMR Peaks		
Peak	Proton Shift	Carbon Shift
1	2.749	21.38
2	1.188	10.11

Figure S23. Full ^{1}H-^{13}C HSQC NMR of **2.**

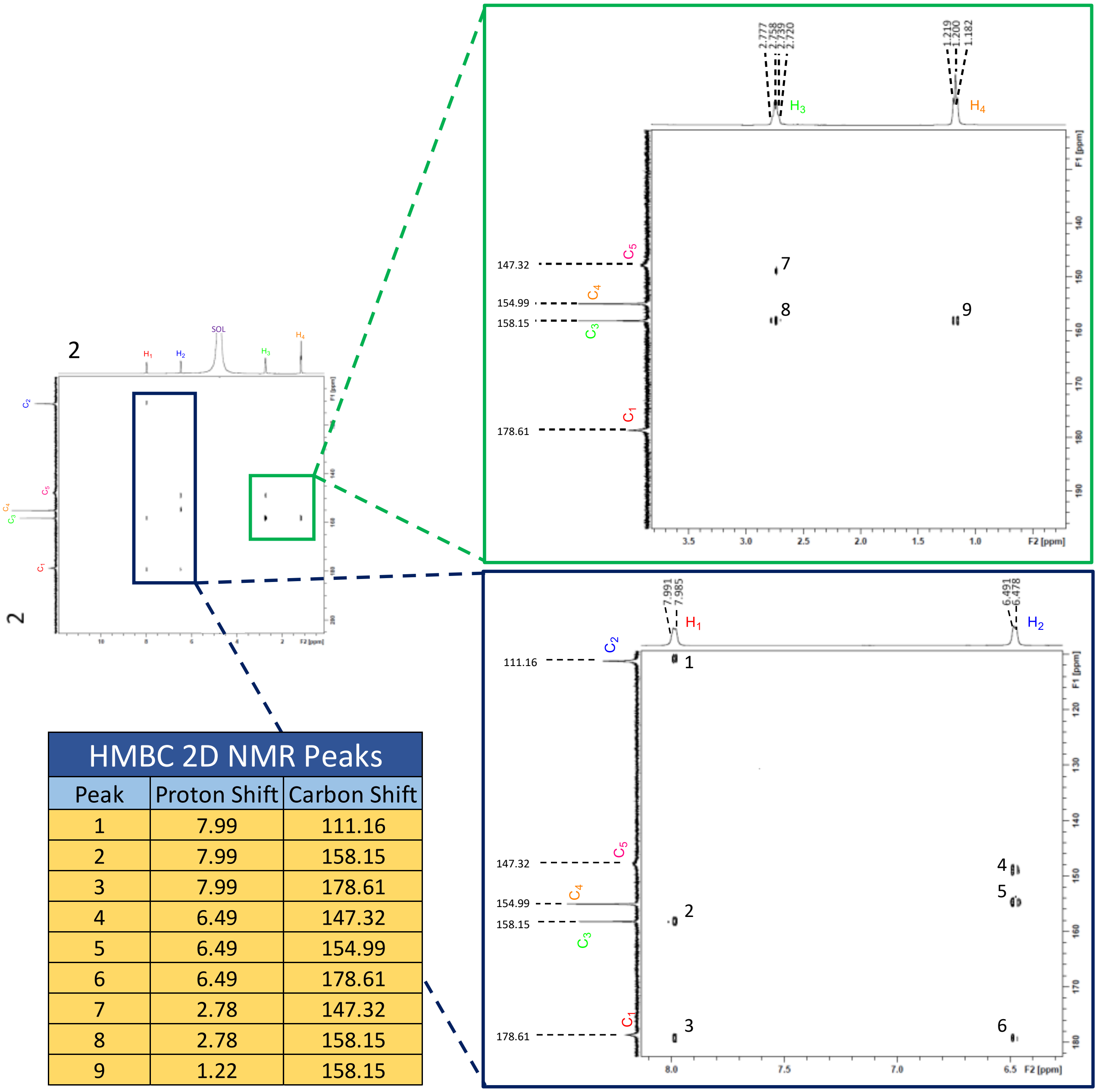

HMBC 2D NMR Peaks		
Peak	Proton Shift	Carbon Shift
1	7.99	111.16
2	7.99	158.15
3	7.99	178.61
4	6.49	147.32
5	6.49	154.99
6	6.49	178.61
7	2.78	147.32
8	2.78	158.15
9	1.22	158.15

Figure S24. Full ^{1}H-^{13}C HMBC NMR of **2.**

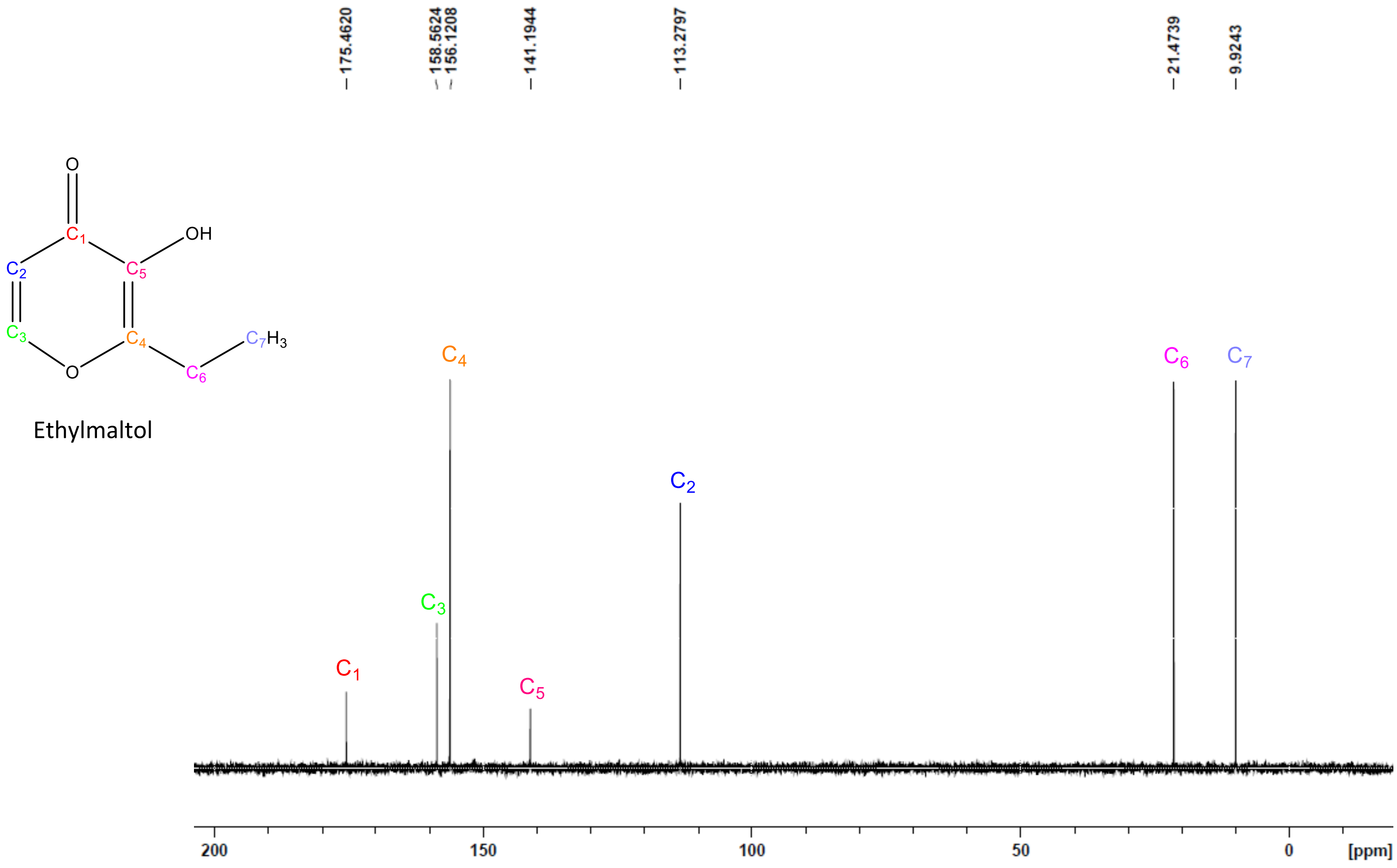

Figure S25. Full ^{13}C NMR of ethylmaltol.

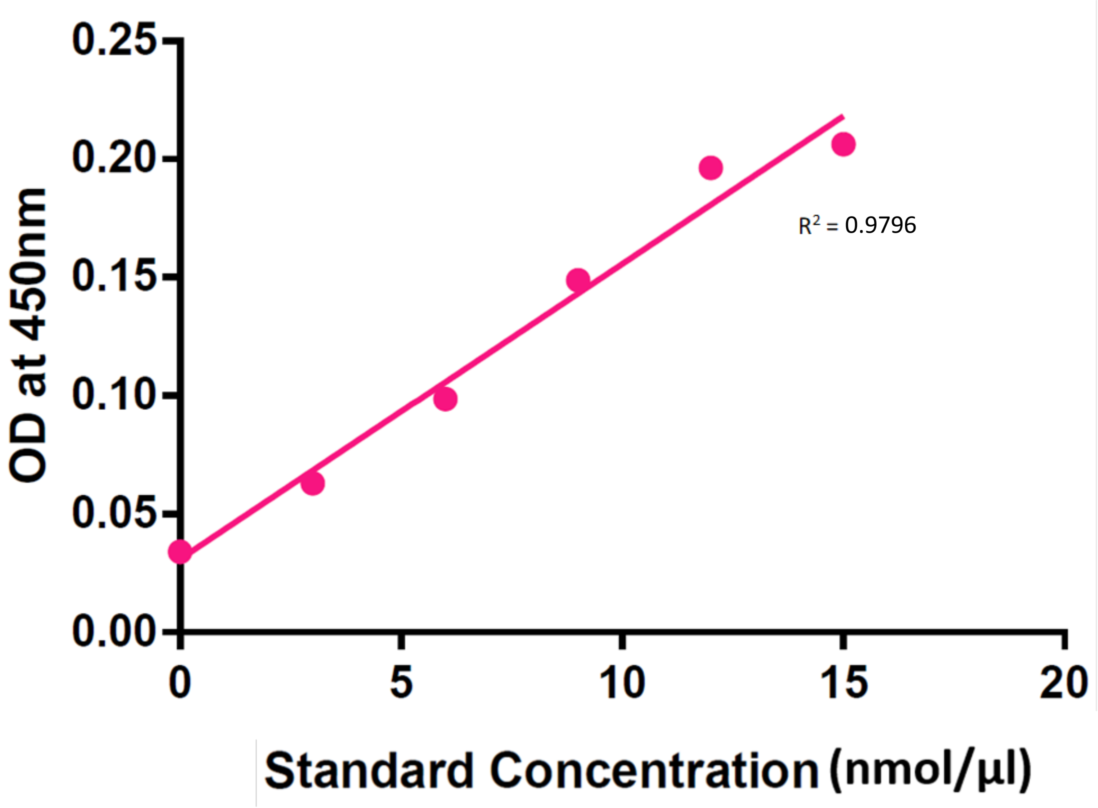

Figure S26. Linear regression of assay standard.
